# Health of neonates born in the maternity hospital in Bern, Switzerland, 1880–1900 and 1914–1922

**DOI:** 10.1371/journal.pone.0289157

**Published:** 2023-08-16

**Authors:** Vivienne Salvisberg, Mathilde Le Vu, Joël Floris, Katarina L. Matthes, Kaspar Staub

**Affiliations:** 1 Institute of Evolutionary Medicine, University of Zurich, Zurich, Switzerland; 2 Department of History, University of Zurich, Zurich, Switzerland; 3 Institute of History, University of Bern, Bern, Switzerland; Iran University of Medical Sciences, ISLAMIC REPUBLIC OF IRAN

## Abstract

The identification of factors impeding normal fetal development and growth is crucial for improving neonatal health. Historical studies are relevant because they show which parameters have influenced neonatal health in the past in order to better understand the present. We studied temporal changes of neonatal health outcomes (birth weight, gestational age, stillbirth rate) and the influence of different cofactors in two time periods. Moreover, we investigated particularly neonatal health in the wake of the 1918/19 influenza pandemic. Data were transcribed from the Bern Maternity Hospital and consists of two time periods: A) The years 1880, 1885, 1890, 1895 and 1900 (N = 1530, births’ coverage 20%); B) The years 1914–1922 (N = 6924, births’ coverage 40–50%). Linear regression models were used to estimate the effect of birth year on birth weight, and logistic regression models to estimate the effect of birth year and of the exposure to the pandemic on premature birth, stillborn and low birth weight (LBW). Mean birth weight increased only minimally between the two datasets; whereas, in the years 1914–1922, the preterm birth and stillbirth rates were markedly reduced compared with the years 1880–1900. Sex, parity, gestational age and maternal age were significantly associated with birth weight in both time periods. The probability of LBW was significantly increased in 1918 (OR 1.49 (95% CI 1.00–2.23)) and in 1919 (OR 1.55 (95% CI 1.02–2.36)) compared to 1914. Mothers who were heavily exposed to the influenza pandemic during pregnancy had a higher risk of stillbirth (OR 2.27 (95% CI 1.32–3.9)). This study demonstrated that factors influencing neonatal health are multifactorial but similar in both time periods. Moreover, the exposure to the 1918/19 pandemic was less associated with LBW and more associated with an increased risk of stillbirth. If this trend is confirmed by further studies, it could indicate some consistency across pandemics, as similar patterns have recently been shown for COVID-19.

## Introduction

Currently, impaired neonatal health remains an ongoing cause for concern for public health worldwide [1]. Identifying determinants that impede normal development and growth is crucial for ameliorating neonatal health overall [2]. An important tool for studying impaired fetal growth is the assessment of anthropometric parameters of newborn infants [3]. As indirect reference values, these parameters allow conclusions to be drawn regarding the intrauterine environment, maternal nutritional status and living standards, as well as predictions of newborn postnatal health and long-term health outcomes (the fetal origin of adult disease hypothesis) [4, 5].

Neonatal body dimensions are determined by various factors, such as genetics of the fetus (e.g., sex) [6] and maternal characteristics, which include genetics, age, height, parity (number of previous pregnancies), pre-pregnancy weight and body mass index, as well as maternal socioeconomic status, level of education, health (e.g., infection) and lifestyle (e.g., cigarette smoking) [7–13]. It is estimated that nongenetic maternal and environmental factors account for 60–70% of the variation in birth weight [14]. In contrast to adult height, birth weight is a more direct measure of nutritional status at the time of measurement (as it mirrors the inherited, biological and environmental influences over nine months of gestation) [5].

Regarding infection, there is a body of literature showing that in utero exposure to influenza has, depending on the trimester, a negative effect on premature births and thus, on neonatal health [15–17]. Studies have further shown that suboptimal maternal nutritional levels, especially third trimester exposure to malnutrition [18], adversely affect fetal growth. Sufficient nutrition and access to adequate health care are dependent on the socioeconomic environment during pregnancy, and recent studies have reported negative influences of economic downturns on neonatal health outcomes [19]. While studies on the effect of the 1918/19 influenza pandemic ("Spanish flu") on birth weight and preterm birth are rare, there is a relatively large demographic literature on the effect of the "Spanish flu" on stillbirths in the USA, Scandinavian countries and Japan [20–25].

The changes in the size, shape, capability, and longevity of the human body since the 19^th^ century reflect both economic improvement of the standard of living and demographic changes [26]. During pregnancy and birth, the health status of females is particularly important for a population’s well-being and development because their nutrition and health status also affects the health and wealth status of the next generation(s) [27–29].

The first descriptive studies on newborn size date back to the 19^th^ century [30, 31]. There was a veritable boom in studies during World War 1 and afterward [32, 33]. In the 1970s and 1980s, initial overviews were published that highlighted the strong association between economic development and birth weight [34]. In anthropometric history, birth weight has still attracted much less attention than height or body mass index. More studies based on maternity hospital data from various cities were conducted in the 1980s and 1990s [5, 31, 35–40]. In recent years, the indicator has again received more scientific attention, which is probably also due to its important role in the intergenerational transmission of wealth and health [5, 41]. Attention is also drawn, quite appropriately, to open questions and inconsistencies related to birth weight as an indicator for living standards [42–44]. The few related studies suggested a particular lack of synchrony between trends in size at birth and increasing trends in height at other ages, as size at birth has been shown to remain stable (e.g., in the USA) [42] or to only increase during the first half of the 20^th^ century (e.g., in Switzerland, Germany or Norway), while adult height increased [40, 45–47].

However, the conclusion that birth weights have been relatively stable over the past 150 years has been reached by comparing means from the late 19^th^/early 20^th^ centuries with modern means [42–44]. Very recently, researchers have also begun to include other anthropometric indicators of neonatal health in the analyses (beyond birth weight) [2, 48]. In Switzerland, birth registers of maternity hospitals in Basel and Bern have proven to be very useful sources of data. Recent studies have thus examined changes in neonatal health during the World War 1 in Basel or selection effects for the same period in Bern [2, 49, 50]. Switzerland is interesting from an international perspective because it was neutral during World War I but the nutritional status of its population was nevertheless affected, although to a lower extent than among neighboring countries [51]. However, information on birth weight from before 1910 and especially at the end of the 19th century is scarce and has not yet been closely studied.

Therefore, the aim of this study, was to analyse for the first time two time-shifted samples from an identical data source, the maternity hospital in Bern: an early sample (1880–1900), when only approximately 20% of births occurred in maternity hospitals, and a later sample (1914–1922), when the coverage was higher (ca. 40–50%). We studied and compared the association between birth weight and different cofactors in the two time periods. Furthermore, we investigated whether there were temporal changes in terms of neonatal health (birth weight, gestational age, stillbirth rate) and particularly if there were neonatal health changes in the wake of the 1918–1919 influenza pandemic in Switzerland, a country that was severely affected by the 1918/19 pandemic.

## Materials and methods

### Data

The data were transcribed from the original birth records of the maternity hospital in Bern (state archive inventory numbers StaBE BB 2.4.151–173). Access to the over 100 years old individual data was allowed by the Staatsarchiv Bern. After transcribing and data cleaning, the data have been fully anonymized. No ethics approval was required from the Canton of Bern for comparable studies by the same group of authors using these data collected over 100 years ago with an expired protection period. More detailed information about the canton of Bern and the maternity hospital can be found in the S1 File. The dataset used in this paper consists of two parts: A) an older part for the years 1880, 1885, 1890, 1895 and 1900 (with 5-year gaps between the years, an annual data set was not yet possible within the given project framework); B) a part for the World War I and “Spanish flu “period as well as the period that shortly followed (1914–1922, continuous).

These records in both datasets include the following individual information for all deliveries that occurred at the hospital in the corresponding years: maternal age, place of residence (categorized into “urban” (the cities of Bern, Biel, and Thun) and “rural”), parity (categorized into 1, 2, 3 and more than 3), birth date of the newborn, infant sex, stillbirth (yes or no), and the infant’s birth weight [52]. In the dataset for the years 1880–1900, the following variables are also provided: qualitative information on maternal body height (categorized into short, medium, tall), maternal body shape (categorized into weak, medium, strong) and nutritional status of the mother (categorized into undernourished or normal), occupation of the mother, and age at menarche. In the dataset covering the years 1914–1922, the following variables are given: insurance status (yes or no) and marital status of the mother (married or not married). These variables can be considered a rough proxy for socioeconomic background in the absence of occupation or educational level [49]. Quantitative information on maternal height and information on the fathers’ occupation were not available for the entire series. We calculated gestational age in weeks as the delta between the date of birth of the newborn and the mother’s indication of when her last menstrual period occurred. When only an estimation of the time of the last menstruation was given (e.g., beginning, middle or end of a month), we used the 5th, 15th and 25th days of a respective month to calculate gestational age.

To estimate the possible impact of the 1918/19 pandemic on neonatal health, we proceeded as follows: individual influenza-like illness symptom (ILI) information (and not confirmed influenza infection) was available (n = 40 cases with ILI symptoms around the time of childbirth). However, this individual sample was presumably small and imprecise. For that reason, we also estimated on an ecological level the flu intensity that the individual women were potentially exposed to during pregnancy. The weekly reported ILI cases from 1918/19 were transcribed from the State Archives of the Canton of Bern using the reported number of ILI cases in the city of Bern (S1 Fig in S1 File). These data have recently been published and discussed in another study [53]. To estimate potential influenza exposure on ecological level, the sum of ILI cases to which each woman was potentially exposed throughout her pregnancy was calculated and normalized between all women (0 = woman had no ILI exposure, 1 = woman had the highest ILI exposure). Furthermore, we estimated the potential exposure during each trimester separately to examine the effects of ILI on neonatal health in the respective trimester.

### Statistical analysis

Because of the temporal gap between the datasets, the slightly different additional variables, and the different coverage of all births in Bern in the two datasets, both datasets were analyzed and presented separately.

#### 1880–1900

Linear regression models were used to estimate the effect of birth year on birth weight, first unadjusted and second adjusted for variables that may have an influence on birth weight, namely sex, parity, gestational age, birth month, maternal height, nutritional status, maternal body shape, maternal age, age at menarche and urbanicity. Since occupation and parity are highly interdependent, two separate regressions were performed with each variable.

#### 1914–1922

Linear regression models were used to estimate the effect of birth year on birth weight, first unadjusted and second adjusted for variables that may have an influence on birth weight, namely sex, parity, gestational age, birth month, maternal age, marital status, insurance status and urbanicity. Unfortunately, we do not have any information on the body height of the mother, which is usually associated with birth weight. We performed a time series analysis of time of birth in calendar weeks to show temporal trends of birth weights and gestational age. A seasonal decomposition to decompose into seasonal, trend and random fluctuation (remainder) components using moving averages and additive seasonal components was performed [54]. In addition, structural break points were tested.

To estimate the effect of year of birth on preterm birth (> = 38 weeks vs < 38 weeks), stillbirth (alive vs stillborn) and low birth weight (LBW <2500 g vs > = 2500g) rates, we used a logistic regression model adjusted for sex, parity, birth month, maternal age, marital status, insurance status and urbanicity. In the model for preterm birth and LBW, stillbirth cases were excluded. The stillbirth model was additionally adjusted for gestational age. In addition, the effect of flu intensity on preterm birth, stillbirth and LBW rates was estimated and adjusted for the cofactors described above. Because year and flu intensity are highly correlated, year is not included in the latter model. In addition, this analysis is performed for each trimester individually. Furthermore, we reduced the dataset to the period in which ILI symptoms were documented (09.07.1918–02.02.1919, N = 40) and descriptively compared whether there were differences in birth weights between women with and without documented influenza-like illness symptoms. Due to the small sample size, statistical tests were not performed.

All statistical analyses were performed using R Version 4.2.2. The R package “dpylr” [55] was used for data manipulation and “ggplot2” [56] to produce all figures.

## Results

### Temporal changes

The sample used for the analysis of the 1880–1900 data set comprised N = 1,530 births (85.7% of the initial data) and N = 6,924 births for the 1914–1922 dataset (89.8% of the initial data). The sample size ranged between N = 275 (1880) and N = 829 (1917). The crude annual mean birth weight varied only very slightly among the years, ranging between 3058.48 gr (SD 542.53) in 1895 and 3172.66 gr (SD 535.76) in 1921 (Tables 1 and 2). The smoothed birthweight distribution plots by year in S2 Fig in S1 File show a tendency toward symmetry. Compared with 1880–1900, the stillbirth rate decreased markedly to 3.3–6.4% in 1914–1922. The proportion of births with a gestational age <38 weeks fell to approximately 13–16% from 1914 onward, with the exception of the years 1917 and 1918, which show the higher values (20.5% and 18.5%). The distribution of all co-factors between the years can be found in S3-S6 Figs in S1 File.

**Table 1:** Descriptive characteristics of the years 1880–1900 (SD = Standard deviation).

	1880 (N = 275)	1885 (N = 315)	1890 (N = 282)	1895 (N = 317)	1900 (N = 341)
**Birthweight (g)**					
Mean (SD)	3119.9 (464.3)	3119.0 (516.9)	3095.0 (528.8)	3058.5 (542.1)	3144.8 (577.7)
Range	1010–4300	1300–4700	1300–4390	1000–4640	1300–5580
					
**Sex**					
missing	1	1	0	0	0
male	145 (52.9%)	177 (56.4%)	149 (52.8%)	147 (46.4%)	172 (50.4%)
female	129 (47.1%)	137 (43.6%)	133 (47.2%)	170 (53.6%)	169 (49.6%)
					
**Parity**					
missing	0	0	0	2	1
1	97 (35.3%)	154 (48.9%)	113 (40.1%)	125 (39.7%)	127 (37.4%)
2	58 (21.1%)	77 (24.4%)	61 (21.6%)	67 (21.3%)	73 (21.5%)
3	41 (14.9%)	25 (7.9%)	31 (11.0%)	41 (13.0%)	34 (10.0%)
>=4	79 (28.7%)	59 (18.7%)	77 (27.3%)	82 (26.0%)	106 (31.2%)
					
**Maternal age**					
Mean (SD)	28.6 (6.7)	27.1 (6.1)	28.6 (6.7)	28.4 (6.5)	27.9 (5.9)
Range	17–48	18–46	17–47	17–45	17–45
					
**Stillborn**					
No	247 (89.8%)	286 (90.8%)	258 (91.5%)	287 (90.5%)	306 (89.7%)
Yes	28 (10.2%)	29 (9.2%)	24 (8.5%)	30 (9.5%)	35 (10.3%)
					
**Gestation group**					
Normal	218 (79.3%)	241 (76.5%)	239 (84.8%)	258 (81.4%)	274 (80.4%)
Early	57 (20.7%)	74 (23.5%)	43 (15.2%)	59 (18.6%)	67 (19.6%)
					
**City**					
Yes	30 (10.9%)	85 (27.0%)	110 (39.0%)	110 (34.7%)	121 (35.5%)
No	245 (89.1%)	230 (73.0%)	172 (61.0%)	207 (65.3%)	220 (64.5%)
					
**Maternal height**					
missing	10	2	7	11	12
short	26 (9.8%)	40 (12.8%)	27 (9.8%)	56 (18.3%)	75 (22.8%)
medium	229 (86.4%)	180 (57.5%)	213 (77.5%)	201 (65.7%)	202 (61.4%)
tall	10 (3.8%)	93 (29.7%)	35 (12.7%)	49 (16.0%)	52 (15.8%)
					
**Nutritional status**					
missing	7	6	7	15	9
normal	251 (93.7%)	259 (83.8%)	259 (94.2%)	292 (96.7%)	312 (94.0%)
undernourished	17 (6.3%)	50 (16.2%)	16 (5.8%)	10 (3.3%)	20 (6.0%)
					
**Maternal physique**					
missing	11	7	4	13	13
grazile	18 (6.8%)	62 (20.1%)	44 (15.8%)	38 (12.5%)	113 (34.5%)
normal	215 (81.4%)	230 (74.7%)	216 (77.7%)	208 (68.4%)	195 (59.5%)
strong	31 (11.7%)	16 (5.2%)	18 (6.5%)	58 (19.1%)	20 (6.1%)
					
**Occupation**					
missing	32	14	10	16	34
Housewifes	57 (23.5%)	64 (21.3%)	117 (43.0%)	129 (42.9%)	131 (42.7%)
Agricultural workers	64 (26.3%)	35 (11.6%)	9 (3.3%)	13 (4.3%)	19 (6.2%)
Domestic service	72 (29.6%)	121 (40.2%)	114 (41.9%)	109 (36.2%)	80 (26.1%)
(Factory) Workers	19 (7.8%)	36 (12.0%)	17 (6.2%)	31 (10.3%)	48 (15.6%)
Craftswomen	25 (10.3%)	25 (8.3%)	8 (2.9%)	12 (4.0%)	14 (4.6%)
Higher occupations	0 (0.0%)	4 (1.3%)	1 (0.4%)	0 (0.0%)	4 (1.3%)
Hospitality sector	6 (2.5%)	16 (5.3%)	6 (2.2%)	7 (2.3%)	11 (3.6%)
					
**Age at 1st menarche**					
missing	20	15	22	29	32
Mean (SD)	16.7 (2.5)	16.4 (2.2)	16.4 (2.3)	16.4 (2.3)	16.4 (2.3)
Range	11–25	12–26	10–26	11–25	11–25

**Table 2:** Descriptive characteristics of the years 1914–1922. (SD = Standard deviation).

	1914 (N=792)	1915 (N=736)	1916 (N=806)	1917 (N=829)	1918 (N=778)	1919 (N=661)	1920 (N=806)	1921 (N=811)	1922 (N=705)
**Birthweight (g)**									
Mean (SD)	3163.7 (526.9)	3130.4 (514.5)	3160.5 (510.2)	3120.8 (526.2)	3128.3 (5123.0)	3168.3 (535.8)	3172.5 (508.7)	3172.7 (535.8)	3169.6 (523.7)
Range	1300–4820	1030–4690	1030–4750	1170–4780	1450–4950	1100–5000	1150–5150	1030–5150	1150–5260
									
**Sex**									
missing	0	0	0	1	0	0	0	1	1
male	407 (51.4%)	368 (50.0%)	426 (52.9%)	431 (52.1%)	393 (50.5%)	351 (53.1%)	419 (52.0%)	433 (53.5%)	363 (51.6%)
female	385 (48.6%)	368 (50.0%)	380 (47.1%)	397 (47.9%)	385 (49.5%)	310 (46.9%)	387 (48.0%)	377 (46.5%)	341 (48.4%)
									
**Parity**									
missing	2	2	5	0	7	0	1	3	1
1	333 (42.2%)	298 (40.6%)	312 (39.0%)	329 (39.7%)	353 (45.8%)	275 (41.6%)	381 (47.3%)	380 (47.0%)	338 (48.0%)
2	171 (21.6%)	147 (20.0%)	197 (24.6%)	170 (20.5%)	146 (18.9%)	131 (19.8%)	172 (21.4%)	150 (18.6%)	141 (20.0%)
3	93 (11.8%)	92 (12.5%)	104 (13.0%)	116 (14.0%)	100 (13.0%)	71 (10.7%)	82 (10.2%)	78 (9.7%)	65 (9.2%)
>=4	193 (24.4%)	197 (26.8%)	188 (23.5%)	214 (25.8%)	172 (22.3%)	184 (27.8%)	170 (21.1%)	200 (24.8%)	160 (22.7%)
									
**Maternal age**									
Mean (SD)	27.4 (6.3)	27.9 (6.5)	27.7 (6.6)	27.8 (6.3)	27.4 (6.1)	27.8 (6.2)	27.3 (6.4)	28.0 (6.5)	27.7 (6.2)
Range	16–46	16–46	14–49	16–47	15–47	16–46	14–50	16–49	14–45
									
**Stillborn**									
No	754 (95.2%)	705 (95.8%)	779 (96.7%)	799 (96.4%)	742 (95.4%)	619 (93.6%)	774 (96.0%)	773 (95.3%)	664 (94.2%)
Yes	38 (4.8%)	31 (4.2%)	27 (3.3%)	30 (3.6%)	36 (4.6%)	42 (6.4%)	32 (4.0%)	38 (4.7%)	41 (5.8%)
									
**Gestation group**									
Normal	673 (85.0%)	617 (83.8%)	681 (84.5%)	659 (79.5%)	634 (81.5%)	569 (86.1%)	694 (86.1%)	693 (85.5%)	597 (84.7%)
Early	119 (15.0%)	119 (16.2%)	125 (15.5%)	170 (20.5%)	144 (18.5%)	92 (13.9%)	112 (13.9%)	118 (14.5%)	108 (15.3%)
									
**City**									
Yes	429 (54.2%)	378 (51.4%)	444 (55.1%)	446 (53.8%)	397 (51.0%)	340 (51.4%)	416 (51.6%)	424 (52.3%)	363 (51.5%)
No	363 (45.8%)	358 (48.6%)	362 (44.9%)	383 (46.2%)	381 (49.0%)	321 (48.6%)	390 (48.4%)	387 (47.7%)	342 (48.5%)
									
**Insurance status**									
missing	0	0	0	0	0	0	0	0	0
not insured	765 (96.6%)	708 (96.2%)	782 (97.0%)	799 (96.4%)	736 (94.6%)	633 (95.8%)	759 (94.2%)	761 (93.8%)	651 (92.3%)
insured	27 (3.4%)	28 (3.8%)	24 (3.0%)	30 (3.6%)	42 (5.4%)	28 (4.2%)	47 (5.8%)	50 (6.2%)	54 (7.7%)
									
**Marital status**									
missing	0	0	0	0	0	0	0	0	0
married	557 (70.3%)	522 (70.9%)	601 (74.6%)	621 (74.9%)	574 (73.8%)	507 (76.7%)	594 (73.7%)	612 (75.5%)	534 (75.7%)
not married	235 (29.7%)	214 (29.1%)	205 (25.4%)	208 (25.1%)	204 (26.2%)	154 (23.3%)	212 (26.3%)	199 (24.5%)	171 (24.3%)

**Fig 1:**
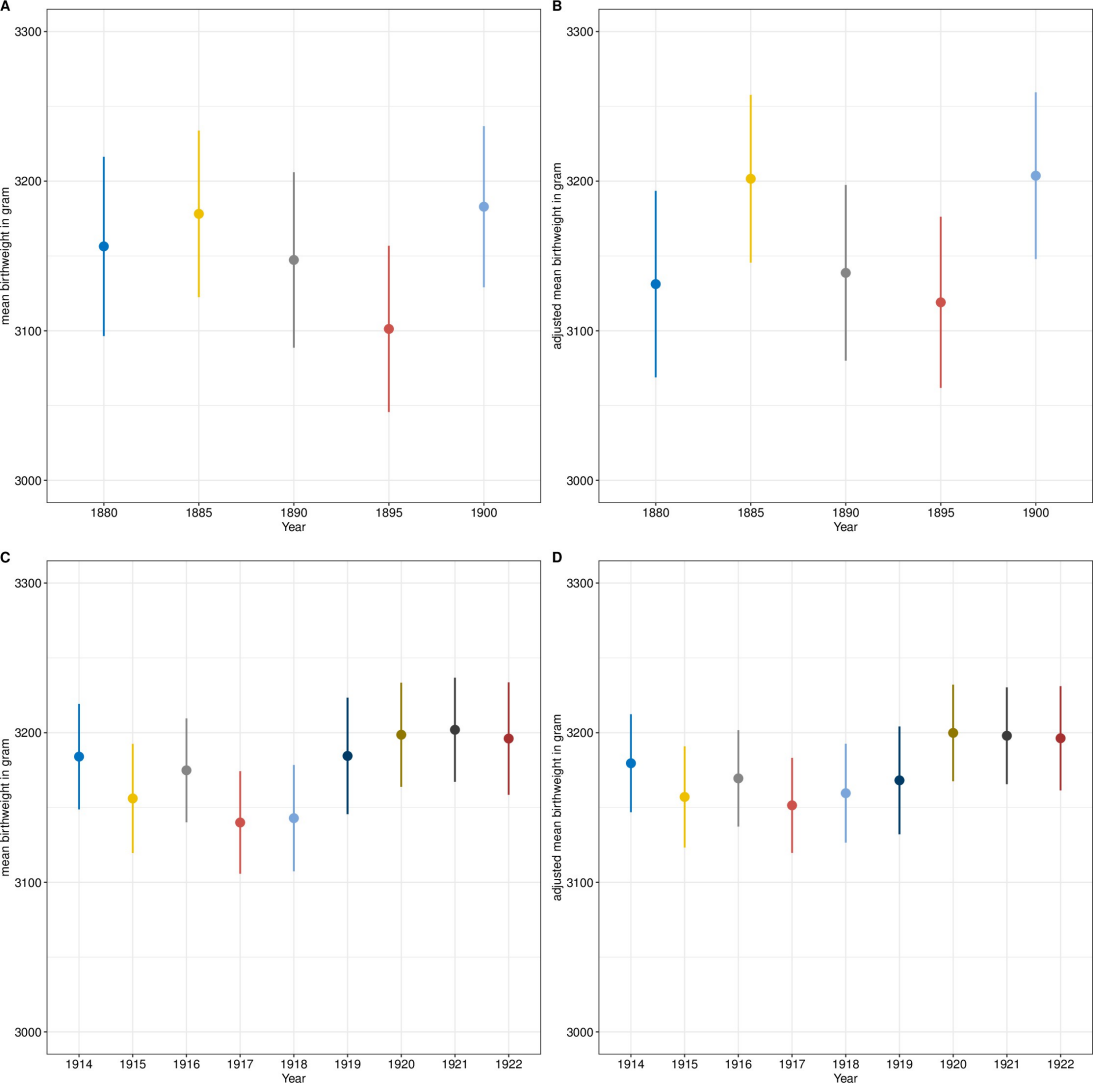
Estimated mean birth weight and 95% Confidence Interval (CI). A) 1880–1900 unadjusted, B) 1880–1900 adjusted for sex, gestational age, birth month, maternal height, nutritional status, maternal body shape, maternal age, age at first menarche and urbanicity, C)1914–1922 unadjusted, D) 1914–1922 adjusted for sex, parity, gestational age, birth month, maternal age, marital status, insurance status and urbanicity.

The annual mean birth weight of the unadjusted and the adjusted regressions are shown in Fig 1A and 1B. There was little change in birthweight between 1880 and 1900; at most, the adjusted mean birth weight was slightly increased in 1900 compared with the previous years (72.45 (95% CI (confidence interval) 12.9–157.8)). The influence of other cofactors can be seen in Table 3 (unadjusted results in S3 Table in S1 File). Because parity and occupation are highly interdependent, two separate regressions were performed, each with one variable, but only the results of adjusting for parity are shown (results for occupation are shown in S4 Table in S1 File). However, the results of the two models are very similar. Females had a lower birth weight than males (-103.09 (95% CI -154.01–-52.16), as did preterm births (-260.33 (95% CI -328.77–-191.9)). The higher the parity (parity > = 4 compared with parity = 1 339.69 (95% CI 255.53–423.85)) was, the higher the birth weight Furthermore, there is a slight tendency for children of short mothers to have a lower birth weight.

**Table 3:** Adjusted results of linear regression of birth weight for 1880–1900. The results were mutually adjusted for all variables. β describes the regression coefficient and 95%CI the 95% confidence interval.

	β (95%CI)
**Year of birth**	
1880*	-
1885	70.38 (-14.31–155.06)
1890	7.5 (-78.08–93.08)
1895	-12.2 (-96.61–72.2)
1900	72.45 (-12.9–157.8)
**Sex**	
Male *	-
Female	-103.09 (-154.01–-52.16)
**Parity**	
1*	-
2	133.17 (65.21–201.14)
3	223.02 (133.25–312.79)
>=4	339.69 (255.53–423.85)
**Gestational age**	
normal	-
preterm	-260.33 (-328.77–-191.9)
**Birthmonth**	
1*	-
2	-48.83 (-171.85–74.18)
3	-49.92 (-174.09–74.25)
4	1.58 (-121.4–124.56)
5	-30.51 (-149.8–88.78)
6	-15.91 (-138.01–106.19)
7	-57.85 (-185.6–69.9)
8	-29.75 (-153.48–93.97)
9	-6.77 (-130.42–116.87)
10	76.48 (-51.5–204.45)
11	-5.99 (-130.7–118.72)
12	99.98 (-15.65–215.6)
**Urbanity**	
urban*	-
rural	-11.91 (-118.3–94.49)
**Maternal height**	
middle*	-
small	-62.62 (-140.08–14.83)
tall	46.81 (-24.39–118.01)
**Malnutrition**	
no*	-
yes	-11.91 (-118.3–94.49)
**Maternal body**	
normal*	-
grazil	-103.38 (-212.4–5.64)
strong	19.1 (-72.18–110.38)
**Age**	5.9 (-5.44–17.24)
**Maternal menarche**	17.82 (-39.7–75.34)

* Reference

The mean birth weight by year (1914–1922) according to the unadjusted and adjusted linear regressions is shown in Fig 1C and 1D. The influence of other cofactors can be seen in Table 4 (unadjusted results in S5 Table in S1 File). The birthweight is slightly decreased in 1917 and 1918 compared to 1914, but not significantly. Female newborns were significantly lighter than male newborns (-148.64 (95% CI -170.73–-126.55)), birth weight increases significantly with increasing parity (parity > = 4 compared with parity = 1 241.36 (95% CI 205.19–277.53)), and children born before 37 weeks of gestation were significantly lighter (-376.67 (95% CI -407.78–-345.55)). In addition, children born to insured mothers were significantly heavier (148.45 (95% CI 95.54–201.36)) than those born to noninsured mothers.

**Table 4:** Adjusted results of linear regression of birth weight for 1914–1922. The results were mutually adjusted for all variables. β describes the regression coefficient and 95%CI the 95% confidence interval.

	β (95%CI)
**Year of birth**	
1914*	-
1915	-22.51 (-69.55–24.53)
1916	-10.11 (-55.98–35.76)
1917	-28.14 (-73.73–17.45)
1918	-20.01 (-66.52–26.5)
1919	-11.41 (-60.05–37.22)
1920	20.29 (-25.68–66.26)
1921	18.4 (-27.66–64.46)
1922	16.71 (-31.14–64.57)
**Sex**	
Male *	-
Female	-148.64 (-170.73–-126.55)
**Parity**	
1*	-
2	131.08 (100.58–161.59)
3	189.6 (151.09–228.12)
>=4	241.36 (205.19–277.53)
**Gestational age**	
normal*	-
preterm	-376.67 (-407.78–-345.55)
**Birthmonth**	
1*	-
2	-40.95 (-95.09–13.18)
3	1.15 (-51.36–53.66)
4	-1.11 (-54.9–52.69)
5	-13.16 (-66.31–39.99)
6	0.11 (-54.28–54.5)
7	-12.87 (-66.19–40.45)
8	12.79 (-41.88–67.46)
9	-1.27 (-55.18–52.63)
10	29.14 (-24.4–82.69)
11	41.33 (-13.19–95.86)
12	-4.36 (-59.41–50.69)
**Urbanity**	
urban*	-
rural	14.87 (-7.47–37.21)
**Married**	
yes*	-
no	-27.38 (-55.28–0.52)
**Insurance**	
no*	-
yes	148.45 (95.54–201.36)
**Age**	-1.83 (-4.04–0.39)

**Fig 2:**
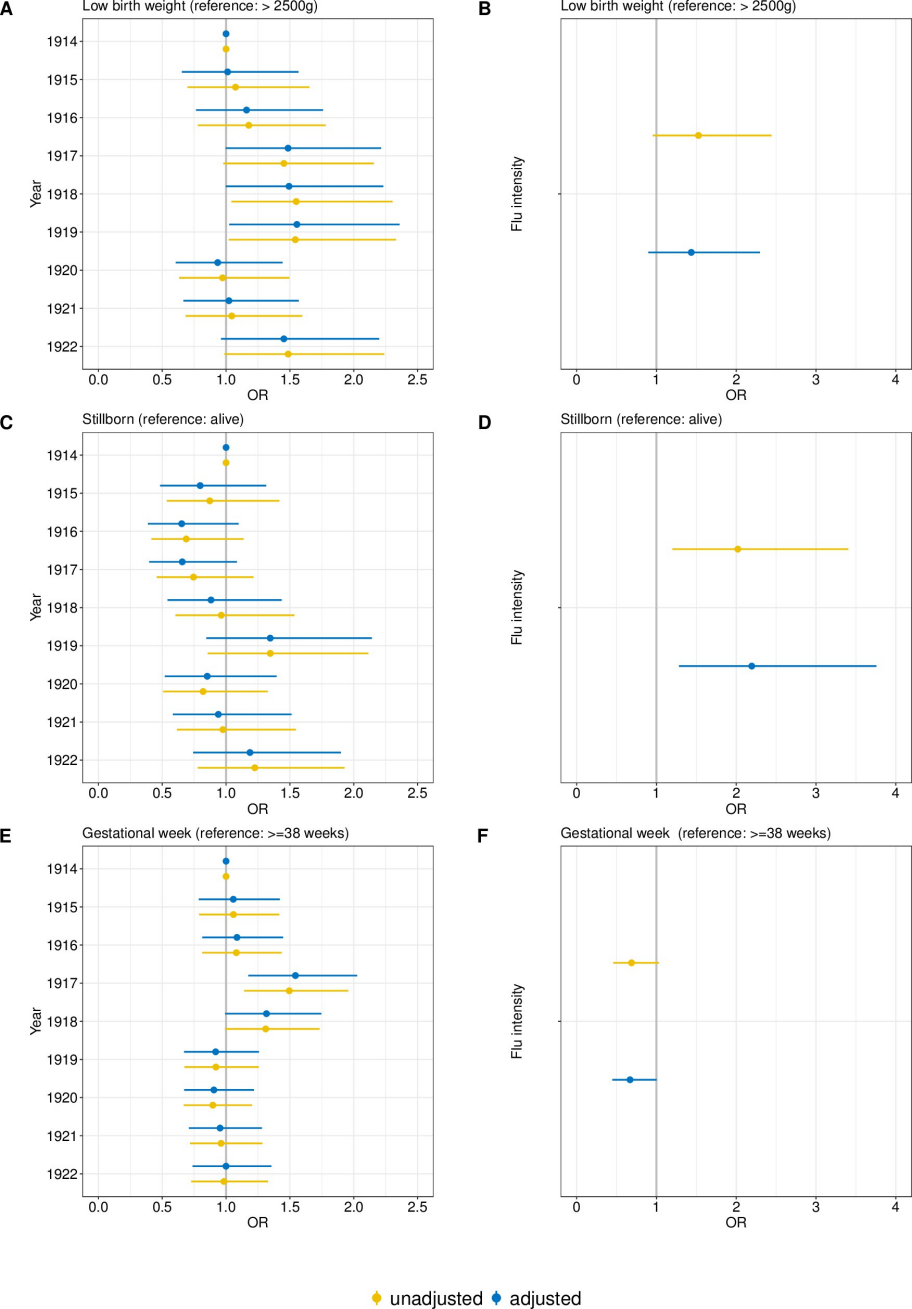
Odds ratios (OR) and 95% Confidence Interval (CI) for different logistical regression models and outcome. A) model for low birth weights and year adjusted for sex, parity, birth month, maternal age, marital status, insurance status and urbanicity, B) model for low birth weights and flu intensity adjusted for sex, parity, birth month, maternal age, marital status, insurance status and urbanicity, C) model for stillborn and year adjusted for sex, parity, birth month, maternal age, marital status, gestational age, insurance status and urbanicity, D) model for stillborn and flu intensity adjusted for sex, parity, birth month, maternal age, marital status, gestational age, insurance status and urbanicity, E) model for gestational age and year adjusted for sex, parity, birth month, maternal age, marital status, insurance status and urbanicity, F) model for gestational age and flu intensity for adjusted for sex, parity, birth month, maternal age, marital status, insurance status and urbanicity.

Only 1919 shows a significant higher risk of LBW (OR 1.55 (95% CI 1.02–2.36) compared to 1914 (Fig 2A). However, 1918 shows a trend towards increased LBW risks (OR 1.49 (95% CI 1.00–2.23)) (Fig 2A). The risk of premature birth increased in 1917 (OR 1.54 (95% CI 1.18–2.03)) compared to 1914, whereas 1918 shows only a tending risk (OR 1.32 (95% CI 0.99–1.75)) (Fig 2E). The OR and CIs are displayed in S6–S8 Tables in S1 File.

#### Pandemic influenza and neonatal health in 1918–1919

The higher the flu exposure was, the higher the risk of stillbirth in the unadjusted (OR 2.02 (95% CI 1.2–3.4)) and adjusted (OR 2.27 (95% CI 1.32–3.9)) model (Fig 2D). The OR and CIs are displayed in S4-S6 Tables in S1 File.

The results of the time series analysis show that there was seemingly no decrease in birth weight during the pandemic in the second half of 1918 but that birth weight increased from week 27 in May 1919 onwards (S7 Fig in S1 File). For gestational age, a slight reduction in 1917 and 1918 (S9 Fig in S1 File) is shown. The stillbirth rate increased from the second half of 1918, with a peak in the first half of 1919 (S8 Fig in S1 File).

Only women who had a higher influenza burden in the first (OR 2.42 (95% CI 1.20–4.87)) and third trimester (OR 2.73 (95% CI 1.42–5.24)) were found to have an increased risk of stillbirth (Table 5).

**Table 5:** Results of the logistic regression for the different neonatal health outcomes and for each trimester. The adjusted models are adjusted for sex, parity, gestational age, birth month, maternal age, marital status, insurance status and urbanicity.

	Flu intensity first trimester	Flu intensity second trimester	Flu intensity third trimester
**Low Birth Weight**	**OR (95%CI)**	**OR (95%CI)**	**OR (95%CI)**
unadjusted	1.46 (0.78–2.74)	2.11 (1.19–3.73)	1.11 (0.59–2.09)
adjusted	1.57 (0.83–3.00)	1.78 (0.98–3.21)	1.09 (0.57–2.09)
** **	** **	** **	** **
**Stillborn**	**OR (95%CI)**	**OR (95%CI)**	**OR (95%CI)**
unadjusted	2.24 (1.13–4.46)	1.75 (0.83–3.66)	2.38 (1.28–4.45)
adjusted	2.42 (1.20–4.87)	1.79 (0.83–3.86)	2.73 (1.42–5.24)
** **	** **	** **	** **
**Gestational weeks**	**OR (95%CI)**	**OR (95%CI)**	**OR (95%CI)**
unadjusted	0.76 (0.44–1.30)	0.88 (0.52–1.47)	0.39 (0.21–0.70)
adjusted	0.71 (0.41–1.22)	0.85 (0.49–1.46)	0.39 (0.21–0.72)

**Fig 3:**
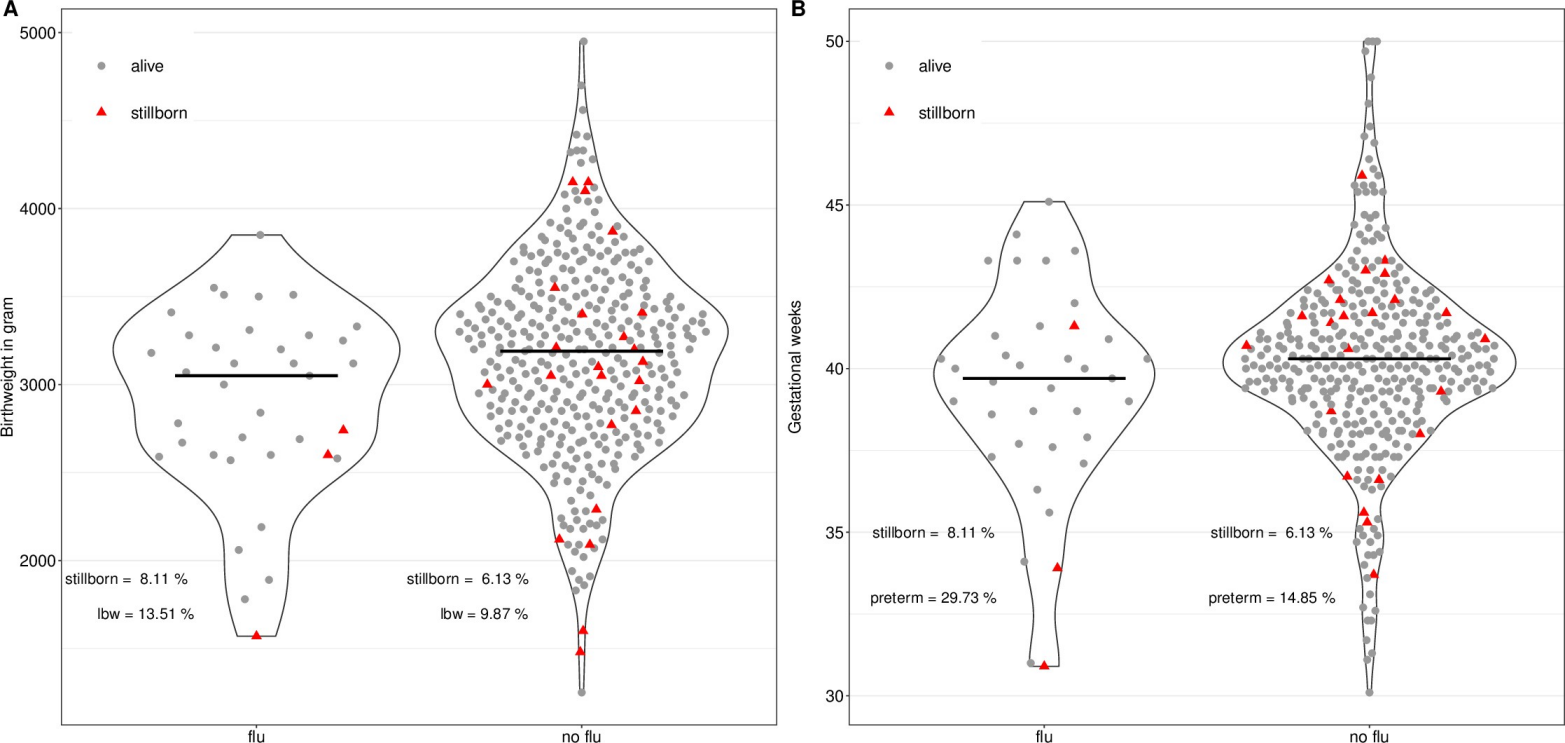
Violin plots grouped by women who had and who did not have ILI symptoms A) Distribution for birthweight, B) Distribution for gestational age.

The boxplots show a slightly reduced birth weight (Fig 3A) and a reduced gestational age (Fig 3B) for women with ILI symptoms compared with women without symptoms. Among those women with ILI, the stillbirth rate was 8.1%, and the low birth weight rate was 13.5%, compared with 6.1% and 9.9%, respectively, in women with no record of ILI symptoms.

## Discussion

We analyzed two datasets of individual data of mothers and their newborns from the maternity hospital of the canton of Bern. Even though comparisons between the two data sets should only be made with caution due to the difference in births coverage, similarities and differences can be seen: Mean birth weight increased only minimally between the two datasets 1880–1900 and 1914–1922. However, in the years 1914–1922, the preterm birth and stillbirth rates were markedly reduced compared with the years 1880–1900. Sex, parity, gestational age and maternal age were significantly associated with birth weight in both datasets. For the period toward the end of the First World War and the 1918 influenza pandemic negative effects on the health of newborns were observed.

Analysis of the cofactors in our two datasets reveals certain comparable patterns that are well known and documented in the literature on historical and modern birth weights, such as the finding that male newborns are heavier than female newborns and that increased parity or maternal age is associated with heavier birth weights [5, 40]. In contrast to other recent studies, for example, on birth weights in the early 20th century in Barcelona [48], we are also able to provide good evidence of the high relevance of the gestational age factor in the data from Bern. Unfortunately, regarding the socioeconomic background of the mothers, maternal occupation was only given in the dataset from 1880–1900. However, the socioeconomic coverage of the data was biased toward the lower class. In the dataset from 1914–1922, however, we still found socioeconomic signals that insured and married mothers gave birth to heavier children.

The mean birth weights we documented for the 1880–1900 are in the same range as those described for other European and US cities at the end of the 19th century (e.g., Bologna, Dublin, Edinburgh, Norway, Utrecht, Vienna) [35, 37, 38, 40, 42, 57]. The same result applies to the influence and importance of essential cofactors such as the sex of the newborn, maternal age, parity, and gestational age. The negative influence of the worsening supply situation due to the course of the war and crop failures in 1916 and 1917 on the health of newborns in 1918 and 1919 has already been shown for the city of Basel [2]. The magnitude of these negative effects is similar for the two cities and is relatively marginal. In contrast to the Basel paper, we were able to account for the possible effect of influenza 1918 and 1919 on neonatal health in the model for Bern. We see no effect of the influenza on birth weights, which is also suggested by a paper on Sweden [17]. 1918–1919 influenza appear to exert its influence primarily through stillbirths. Of course, it is notoriously difficult or perhaps impossible to strictly distinguish between the negative effects of the World War and influenza.

Our main finding concerning the impact of indirect influenza exposure (measured by the intensity of flu cases to which a woman has been exposed) is an increase in the stillbirth rate by a factor of 2.2. This is consistent with the evidence from demographic studies on the population level for the USA, Scandinavian countries or Japan [20–25]. In the United States, it was found that increasing mortality rates from ILI in the population were associated with an increase in neonatal and infant mortality in the late 20^th^ century [49]. Depending on the trimester of exposure, various adverse pregnancy outcomes were also highlighted. Among the women who developed ILI during pregnancy or delivery in the Bern data, stillbirth rates and low birth weight rates increased compared with those in women without a record of ILI. Comparable data from Basel have shown that negative effects in 1918 were influenced not only by the pandemic but also by the increasing malnutrition towards the end of war [2]. Which influence was stronger remains unclear.

Our study has several limitations. When working with historical birth weight data, it is central to discuss the issue of selection bias [5, 40, 42, 44]. The data from 1880–1900 were derived from a time when the maternity hospital was also a welfare institution, and most births occurred at home. One of the explicit purposes of the institutions at that time was to provide poorer and unmarried expectant mothers an opportunity to give birth [58]. By the time of the second dataset from 1914–1922, this had already changed fundamentally; hospital birth was gaining acceptance in the general population, and the purpose of the welfare institution had disappeared from the hospital’s statutes. The coverage of the dataset from 1914–1922 is thus considerably higher than that from 1880–1900 (approx. 40–50% vs. approx. 20%). Furthermore, it must also be noted that the cantonal maternity hospital in Bern was not the only hospital in the city and canton of Bern where mothers delivered [58]. These other hospital births as well as all home births are not represented in our dataset. This limits the temporal comparability of the two data sets. Because we were not able to precisely determine the functional form of the selection and outcome processes, we have refrained from using correction methods [59]. Despite this limitation, especially with regard to cross-temporal comparisons, which underlie all similar analyses, the literature on historical birth weights has demonstrated the potential that such data can provide. At a time of improving living standards and health status since the end of the 19th century, such data on maternal and neonatal health provide an important focal point at a crucial time of intergenerational transmission of health and wealth [5, 40, 42, 44]. The literature on historical birth weights discusses whether mean birth weight (analogous to adult height) increased over the course of the 20th century [42]. Regarding adult height, it has been shown that the secular trend toward an increase in the canton of Bern (city and countryside) started with the birth cohorts from approximately 1890–1900 [60]. Due to the reasons mentioned above, we cannot conclusively answer the question about rising average birth weight based on our maternity hospital data either. We see a very slight increase in mean birth weight between the two datasets, but the extent to which the change in sample composition may explain this remains unclear. Based on the birth records of the Federal Statistical Office from 2021, excluding multiple births and stillbirths, the mean birth weight for the canton of Bern in 2021 is around 3330g. This birth weight is about 100 grams higher than in the years 1880–1922. However, the birth weights of today are difficult to compare with the birth weights of 1880–1922 due to changed living conditions. Moreover, the preterm birth and stillbirth rates decreased significantly between the two datasets. Besides the increasing coverage of the data since the 1880–1900 data, this reduction might be also explained by medical progress in obstetrics, as well as advances in hygiene, general health care, and midwifery in the 1900s [58, 61, 62].

Furthermore, maternal occupation was only documented for the earlier dataset from 1880–1900; for the years1914-1922, these data were no longer available, and marriage status and insurance status were the only proxies for socioeconomic status. Another limitation is that maternal body height, which is an important influencing factor [63, 64], was not always recorded in the Bernese dataset and only as a qualitative variable, which is much less precise than quantitative height. This also applies to other important factors influencing neonatal health (e.g., maternal lifestyle during pregnancy, genetics, stress, exposure to infectious diseases other than influenza), which cannot be investigated in this study. A further limitation is that the information on whether a mother was ill with influenza in 1918 and during which pregnancy trimester is somewhat imprecise in the Bernese source data, which is why we also took the ecological route of a possible exposure of all expectant mothers to supplement this. Nevertheless, the births studied during the pandemic are subject to survival bias. A possible increase in miscarriage risk due to exposure to influenza during the first trimester would have gone unnoticed because it was underreported. Thus, we do not have information on unobserved fetal loss early in pregnancy [49].

## Conclusions

Although the representativeness of our dataset increases across the two observation periods and the results need to be interpreted accordingly, our study shows that the factors influencing neonatal health were similar across the two datasets. The data on influenza infection during pregnancy were too imprecise in this source to be conclusive at the individual level. However, it appears that “Spanish flu” infections were less associated with lower birth weight and more associated with an increased risk of stillbirth. If this trend is confirmed by further studies, it could indicate some consistency across pandemics, as similar patterns have recently been shown for COVID-19 [65].

## Supporting information

S1 Text (TXT)

S1 File (PDF)
