# Supplementary material for: Health of neonates born in the maternity hospital in Bern, Switzerland, 1880–1900 and 1914–1922
Source: PLoS One. 2023 Aug 16;18(8):e0289157. doi: 10.1371/journal.pone.0289157 (PMC10431681; doi:10.1371/journal.pone.0289157)
Supplement: S1 File — (PDF) [file pone.0289157.s002.pdf]

The canton of Bern

The canton of Bern 1880-1925

The canton of Bern is the second largest of the Swiss cantons by surface area, and in 1920, it was also the most populated canton [1]. Topographically, it is located in the Swiss midland and consists of three main regions, the Jura Mountains in the north, the midland, and the alpine areas in the south. The cantonal capital, the city of Bern, is also the seat of the Swiss federal parliament and the Swiss government. Bern was and still is considered to be a typical rural region. At the end of the 19th century, the canton encompassed a dozen smaller towns, but the vast majority of its municipalities were rural and not yet modernized (which remained unchanged until far into the 20th century) [2]

Between 1870 and 1930, all documented indicators of wellbeing and prosperity in the canton of Bern and in Switzerland in general showed a clear improvement, temporarily interrupted only by the first world war (in which Switzerland was not directly involved) and the influenza pandemic in 1918 [3]. Regarding GDP per capita, the canton Bern ranked 20th out of 25 cantons in 1880 and improved to 17th out of 25 in 1920 [64]. In this phase of increasing wealth, marked population growth occurred (from 478,364 in 1880 to 615,916 in 1920), and the degree of urbanization increased as well. In Bern, as elsewhere in Switzerland, due to a first step of globalization in the 1870s in the course of the coupling of the Swiss railroad network to the worldwide transport network, the cheap mass import of food became possible. The fact that from the 1870s onward prices stabilized while wages continued to rise led to an increase in the standard of living for the population overall. The improvement in real income was associated with a decline in the importance of food expenditures in the household budget. In 1830-1875, a working-class family in Switzerland spent approximately 62 percent of its budget on food, while at the beginning of the 20th century, food accounted for 40-50 percent of the total expenditure, and by 1950, it had fallen to 33 percent [4].

Not only do all monetary indicators of living standards trend upward in the canton of Bern for the period under study (1880-1920), but health and demographic indicators trend in the same direction; specifically, life expectancy has increased, infant and child mortality rates have fallen sharply, and the average adult height has increased. In addition to an improved nutritional status of the population, improvements in sanitary conditions and hygiene (e.g., WASH, clean and waste water supply), discourses about hygiene and nutrition, and better social welfare are often listed among the reasons, together with improved housing conditions. This is also reflected in the ongoing epidemiological transition, as the burden of infectious diseases has been greatly reduced during this period [5]

The proportion of women in the labor force relative to the total female population in the canton of Bern slightly increased from 23.9% in 1880 to 25.9% in 1920 (the respective numbers for men were 60.2% and 64.6%) [66]. The sectoral distribution of working women underwent a significant change between 1880 and 1920; specifically, while 44.3% of working women were still employed in the agricultural sector in 1880 (compared with 39.7% in industry and 16.0% in the service sector), the proportion of working women in the service sector in particular doubled until 1920 (32.6%, compared with only 22.2% left in agriculture and 45.2% in industry) (S1 Table). There were striking differences between urban and rural areas and between the centers and the periphery. These shifts reflect the ongoing industrial transition that brought a decline in self-sufficient family-scale farming, as well as the creation of new options for paid work outside the home (e.g., industrialized textile industry and domestic services). However, the proportion of housewives among adult women was high at the beginning of the 20th century, which may have had an effect on the offspring's health outcome due to the accompanying emphasis on motherhood, which seemed to have triggered better attention and improved maternal care during pregnancy and the offspring's infancy (e.g., introduction of reduced working hours for women) [6].

At the beginning of the 20th century, the First World War and especially the years 1917 to 1919 resulted in a marked interruption of the growing prosperity, and the influenza pandemic of 1918 and 1919 aggravated the situation. Although neutral Switzerland was spared military attacks during World War I, it was still severely affected by the length of the war and, in particular, by the Allied blockade and the resulting economic and social difficulties [7]. Switzerland particularly suffered because of its dependency on imports of grain and raw materials [8]. Toward the end of the war, food consumption in Switzerland had fallen to three quarters of its prewar level, and by 1917, the Swiss had approximately 25-30 percent fewer calories at their disposal than before the war. In the city of Bern, the proportion of the total household budget spent on food rose considerably in working-class families as well as in families of civil servants and employees. In working-class families, it increased from 46.9 percent in 1912 to 54.1 percent in 1919, and in families of civil servants and employees, the increase was even somewhat greater (32.4 percent had to be spent on food in 1912, compared with 38.4 percent in 1919). The fact that the food shortage was not as severe as in the warring neighboring countries is probably due in part to the relatively long and artificially low-kept milk price. Milk was the least expensive of all basic foodstuffs. During the first years of the war, milk consumption was still 19 percent higher than that in a normal year and even increased until 1916.

Anthropometric information on the birth weight of newborns in the city of Basel, on the growth monitoring of Bernese schoolchildren and on the body shape of conscripts during enlistment shows that the human body in Switzerland certainly reflected the increasingly deteriorated nutritional situation at the end of the First World War [4]. In the sense of interactions between nutritional status and immune status, the influenza pandemic exacerbated the situation in July/August (wave 1) and from October 1918 (waves 2 and 3). The canton of Berne was hit hard, albeit with regional differences (the cities had particularly high incidences and mortality rates) [1]. The course of the 1918 pandemic in the canton and the city of Bern is displayed in S1 Fig.

The maternity hospital in Bern

The cantonal maternity hospital in the city of Bern ("Frauenspital" or "Kantonale Entbindungs- und Frauenkrankenanstalt") was founded in 1875 [8]. Until the second half of the 19th century, maternity hospitals in general served only to admit women in childbirth. In the 19th century, women from lower socioeconomic positions were hospitalized to give birth because their housing situation did not offer good conditions for childbirth. The wealthier women usually preferred and could afford a home birth and midwives. Because hygiene conditions in hospitals were not favorable until the beginning of the 20th century, hospital births tended to be avoided. However, maternity hospitals underwent a change at the end of the 19th century [9]. With the increasing safety of hospitals and hospital births, the proportion of hospital births compared with home births rose significantly at the beginning of the 20th century. Maternity hospitals in general transformed markedly with the rise of obstetrical and gynecological sciences, together with broad improvements in midwifery. In addition to providing medical care, the maternity hospital of Bern also served as a training hospital for the university and as a midwifery school [9]. In approximately 1915, the maternity hospital of Bern represented the most important gynecological center in the Bernese midland region, and the increasing demand for hospital births and population growth in general triggered an extension of the existing building in 1919.

In S2 Table, a comparison of children born in the Bern maternity hospital (aggregated from the individual hospital data) with all children born in the city and canton of Bern between 1880 and 1925 shows that the relative proportion of all births in the city of Bern that occurred in the maternity hospital increased by 10 percentage points between 1899 and 1901 (from 13.7% to 24.8%) and then stabilized at approximately 20% until around 1908. Then, the proportion of hospital births began to rise significantly to >40% at the start of WW1.During WW1, there was another marked increase in the rate of hospital births, and after WW1, the hospital birth proportion constantly exceeded 50%. If the number of births in the entire canton of Bern is taken as a comparison, the proportions are of course smaller, but the pattern of temporal change is the same.

References

[1] K. Staub *et al.*, “Public health interventions, epidemic growth, and regional variation of the 1918 Spanish flu outbreak in a Swiss Canton and its greater regions,” *Ann. Intern. Med.*, vol. 174, no. 4, pp. 533–539, 2021.

[2] T. Schoch, K. Staub, and C. Pfister, “Social inequality and the biological standard of living: An anthropometric analysis of Swiss conscription data, 1875-1950,” *Econ. Hum. Biol.*, vol. 10, no. 2, pp. 154–173, 2012, doi: 10.1016/j.ehb.2011.05.001.

[3] J. Floris, F. Höpflinger, C. Stohr, R. Studer, and K. Staub, “Wealthier – older – taller: measuring the standard of living in Switzerland since the 19th century,” *Schweizerische Zeitschrift für Geschichte*, vol. 69, no. 2, pp. 207–232, 2019.

[4] K. Staub, “Der vermessene menschliche Körper als Spiegel der Ernährungs- und Gesundheitsverhältnisse am Ende des Ersten Weltkrieges,” in *«Woche für Woche neue Preisaufschläge» Nahrungsmittel-, Energie- und Ressourcenkonflikte in der Schweiz des Ersten Weltkrieges.*, S. D. Krämer D, Pfister C, Ed. Basel: Schwabe Verlag, 2016.

[5] K. Staub, “Grösser - und dicker. Körperhöhe und Body Mass Index im Kanton Bern seit dem 19. Jahrhundert.”

[6] N. Koepke, J. Floris, C. Pfister, F. J. Rühli, and K. Staub, “Ladies first: Female and male adult height in Switzerland, 1770–1930,” *Econ. Hum. Biol.*, vol. 29, 2018, doi: 10.1016/j.ehb.2018.02.002.

[7] C. R. Michael Brendan Schumacher, Kaspar Staub, “Veränderungen des kindlichen Geburtsgewichts und anderer Geburtsparameter im Frauenspital Bern 1916 bis 1920 - eine historisch-statistische Analyse,” 2016.

[8] Joël Floris, Laurent Kaiser, Harald Mayr, Kaspar Staub, and Ulrich Woitek, “Survival of the weakest? Culling evidence from the 1918 flu pandemic,” Zurich, 316, Jan. 2019.

[9] K. S. Nadine Duss, “Wie beeinflusst der Ernährungszustand einer Mutter das Geburtsgewicht ihres Neugeborenen?,” 2015.

Tables and Figures

S1 Table: Occupational composition of the male and female population of the Canton of Bern in the census years 1880-1920 (Source: Swiss Census).

**Men**	**1880**	**1888**	**1900**	**1910**	**1920**
Agriculture	48.9	47.1	41.5	37.3	36.2
Industry	37.4	38.1	42.4	43.8	44.0
Trade	5.4	5.3	6.0	7.5	7.7
Transport	3.1	4.2	5.1	5.8	6.2
Public administration, science & art	4.3	4.5	4.8	5.2	5.6
Personal services	1.0	0.8	0.2	0.4	0.3

**Women**	**1880**	**1888**	**1900**	**1910**	**1920**
Agriculture	44.3	42.5	36.3	23.2	22.2
Industry	39.7	39.1	42.8	46.0	45.2
Trade	9.3	7.9	9.1	18.1	19.0
Transport	0.6	3.7	4.7	1.5	1.9
Public administration, science & art	3.2	5.4	6.3	7.5	7.9
Personal services	2.9	1.3	0.8	3.7	3.8

S2 Table: The total number of births in the canton and city of Bern and the number of births in the maternity hospital in Bern (also relative, expressed as a %) by year.

Year	Births maternity (n)	Births canton (n)	Births city (n)	Perc. Canton (%)	Perc. City (%)
1878	376	18489	1610	2.0	23.4
1879	362	18100	1527	2.0	23.7
1880	333	17423	1487	1.9	22.4
1881	371	18115	1503	2.0	24.7
1882	337	17625	1476	1.9	22.8
1883	384	17746	1514	2.2	25.4
1884	322	17603	1392	1.8	23.1
1885	253	17478	1442	1.4	17.5
1886	345	17603	2275	2.0	15.2
1887	311	17665	2286	1.8	13.6
1888	355	17590	2349	2.0	15.1
1889	334	17559	2366	1.9	14.1
1890	337	17000	2350	2.0	14.3
1891	405	17728	2382	2.3	17.0
1892	418	18133	2457	2.3	17.0
1893	451	18242	2517	2.5	17.9
1894	400	17562	2438	2.3	16.4
1895	380	17885	2565	2.1	14.8
1896	399	18375	2682	2.2	14.9
1897	415	18450	2796	2.2	14.8
1898	472	18903	2943	2.5	16.0
1899	413	18947	3025	2.2	13.7
1900	not available	18939	2946	not available	not available
1901	503	19631	2040	2.6	24.7
1902	480	19311	2044	2.5	23.5
1903	508	18667	2016	2.7	25.2
1904	492	18750	2032	2.6	24.2
1905	518	18504	2088	2.8	24.8
1906	541	18845	2200	2.9	24.6
1907	563	18115	2024	3.1	27.8
1908	548	18287	2065	3.0	26.5
1909	657	18022	2021	3.6	32.5
1910	669	17793	2049	3.8	32.7
1911	687	17053	1976	4.0	34.8
1912	720	17089	1974	4.2	36.5
1913	not available	16688	2038	not available	not available
1914	898	16465	1891	5.5	47.5
1915	812	14602	1693	5.6	48.0
1916	872	14464	1724	6.0	50.6
1917	912	14281	1638	6.4	55.7
1918	835	14055	1556	5.9	53.7
1919	721	14549	1716	5.0	42.0
1920	882	15677	1877	5.6	47.0
1921	875	15624	1874	5.6	46.7
1922	770	14604	1779	5.3	43.3
1923	835	14673	1867	5.7	44.7
1924	819	14054	1646	5.8	49.8
1925	874	13802	1718	6.3	50.9
1926	910	13629	1609	6.7	56.6
1927	905	12862	1466	7.0	61.7
1928	975	12775	1448	7.6	67.3
1929	1038	12540	1517	8.3	68.4
1930	1175	12359	1493	9.5	78.7

S1 Fig: The course of the 1918/1919 influenza pandemic in the city and canton of Bern by calendar week 1914-1922. Top: Total number of deaths (all causes of death); Bottom: Newly reported cases of influenza-like-illness and Weekly Bulletin of the Federal Office of Public Health). The course of the pandemic (including measures taken by the authorities and double summits in the second wave) are analyzed and discussed in detail here: https://www.acpjournals.org/doi/10.7326/m20-6231.

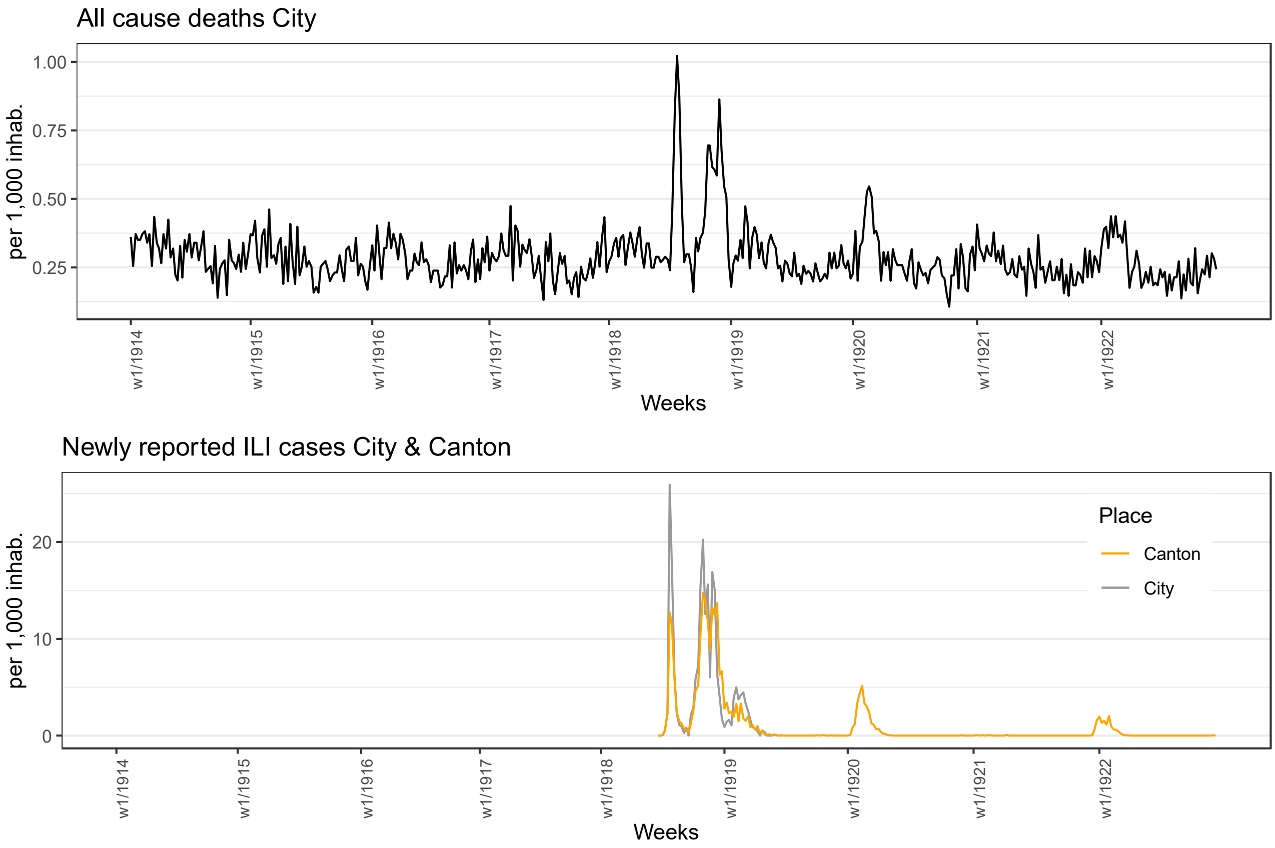

S2 Fig: Birth weight distribution per years for A) 1880 - 1900 and B) 1914 - 1922

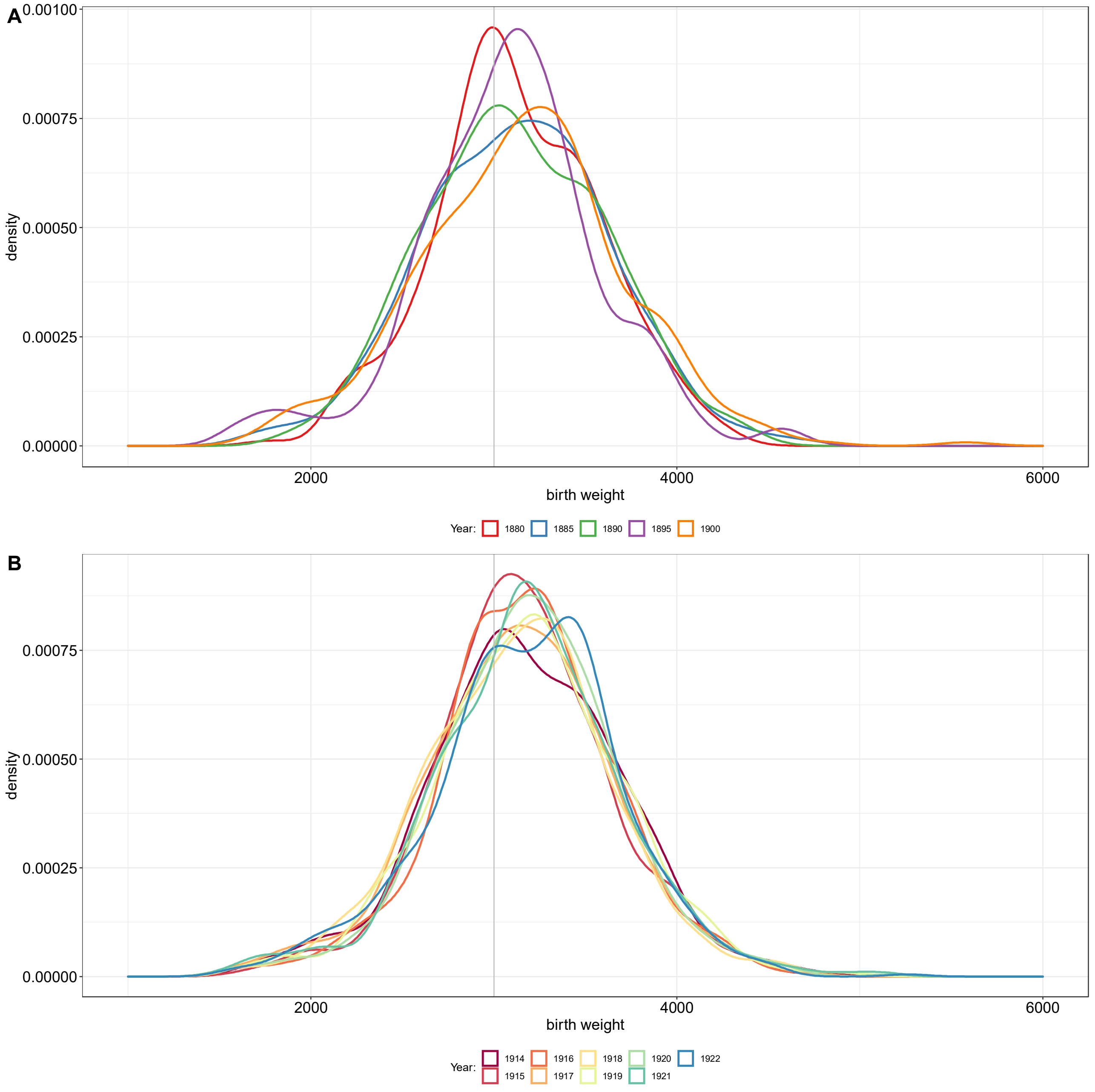

S3 Fig: Violin plots by year for A) birthweights, B) ager of mother, C) gestational age, D) age menarche.

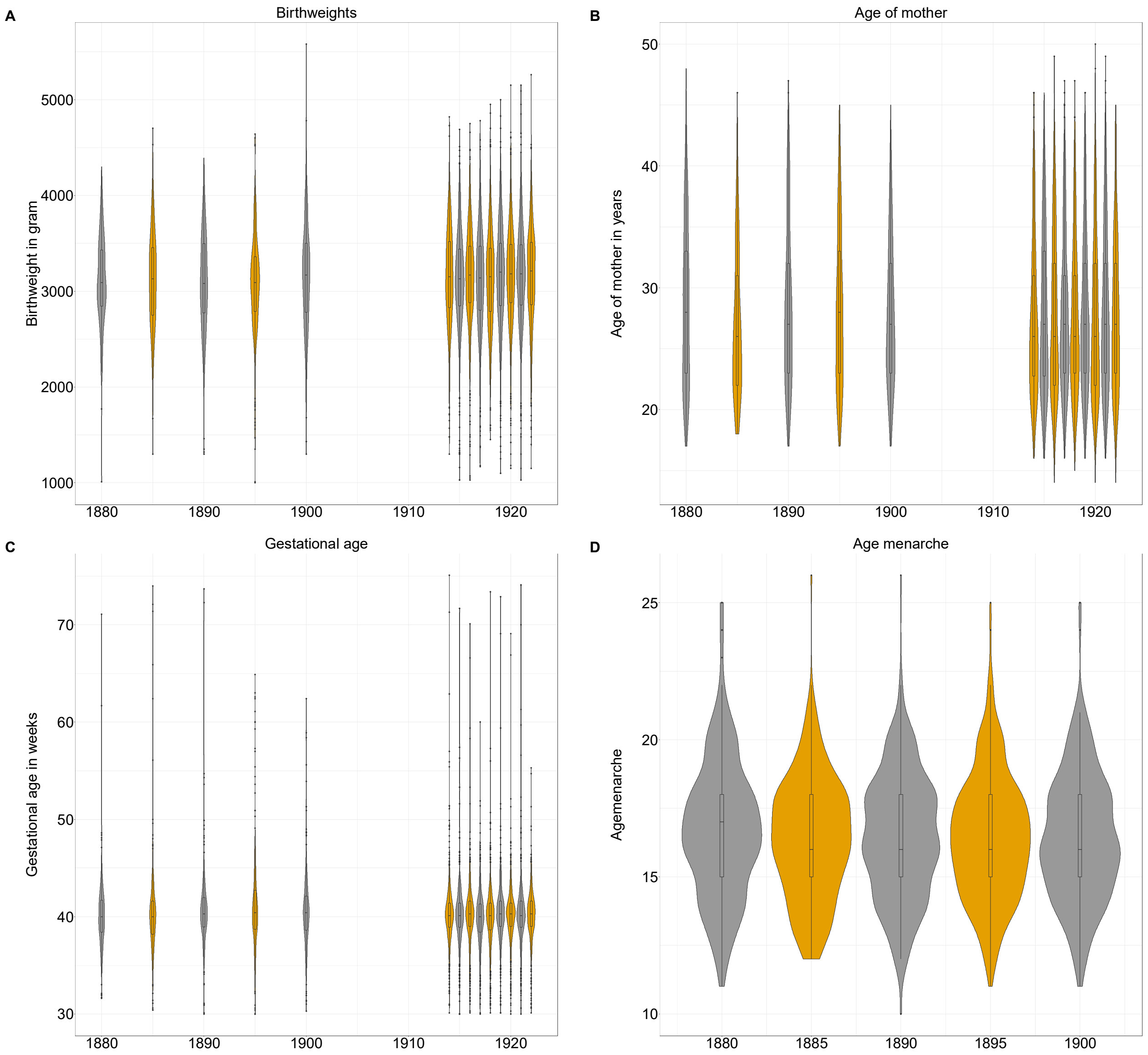

S4 Fig: Barplots by year for A) sex, B) parity, C) gestational age (early <37 weeks, normal >=27 weeks, D) stillborn, E) urbanity, F) having an insurance G) married

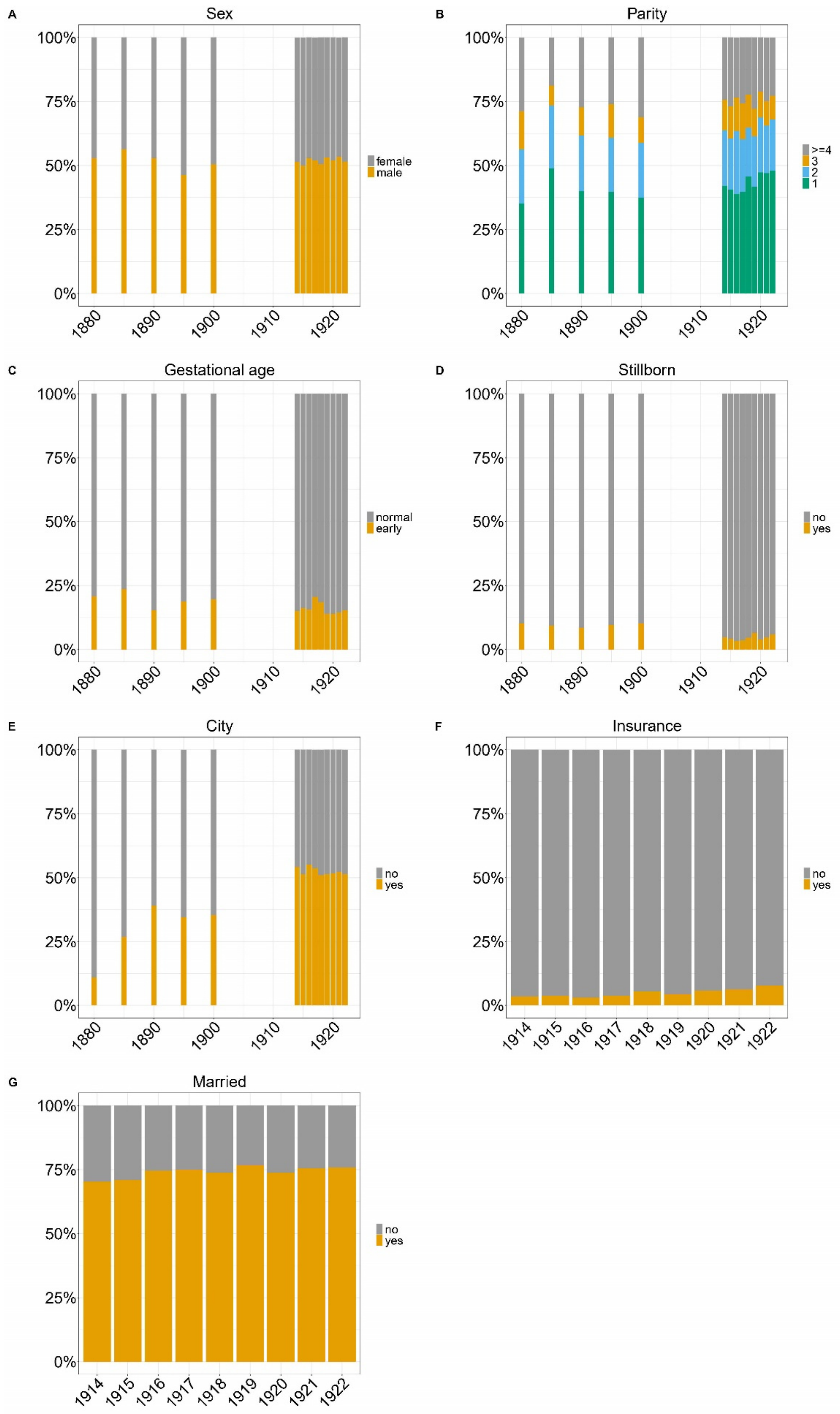

S5 Fig: Barplots for A) birth month B) weekday of birth, C) birth month by year, D) weekday by year

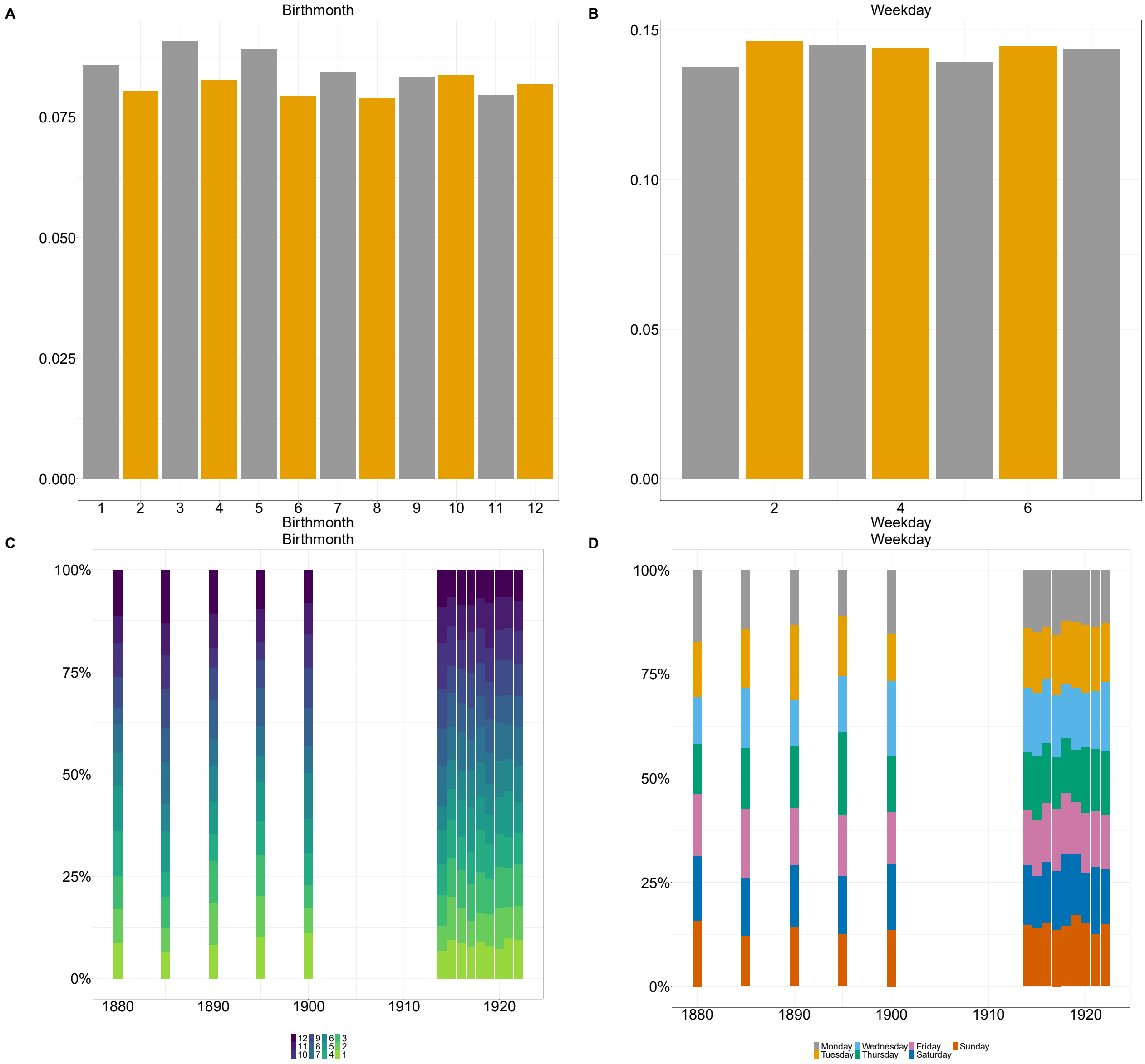

S6 Fig: Barplots by years A) maternal body, B) maternal height, C) malnutrition, D) occupation

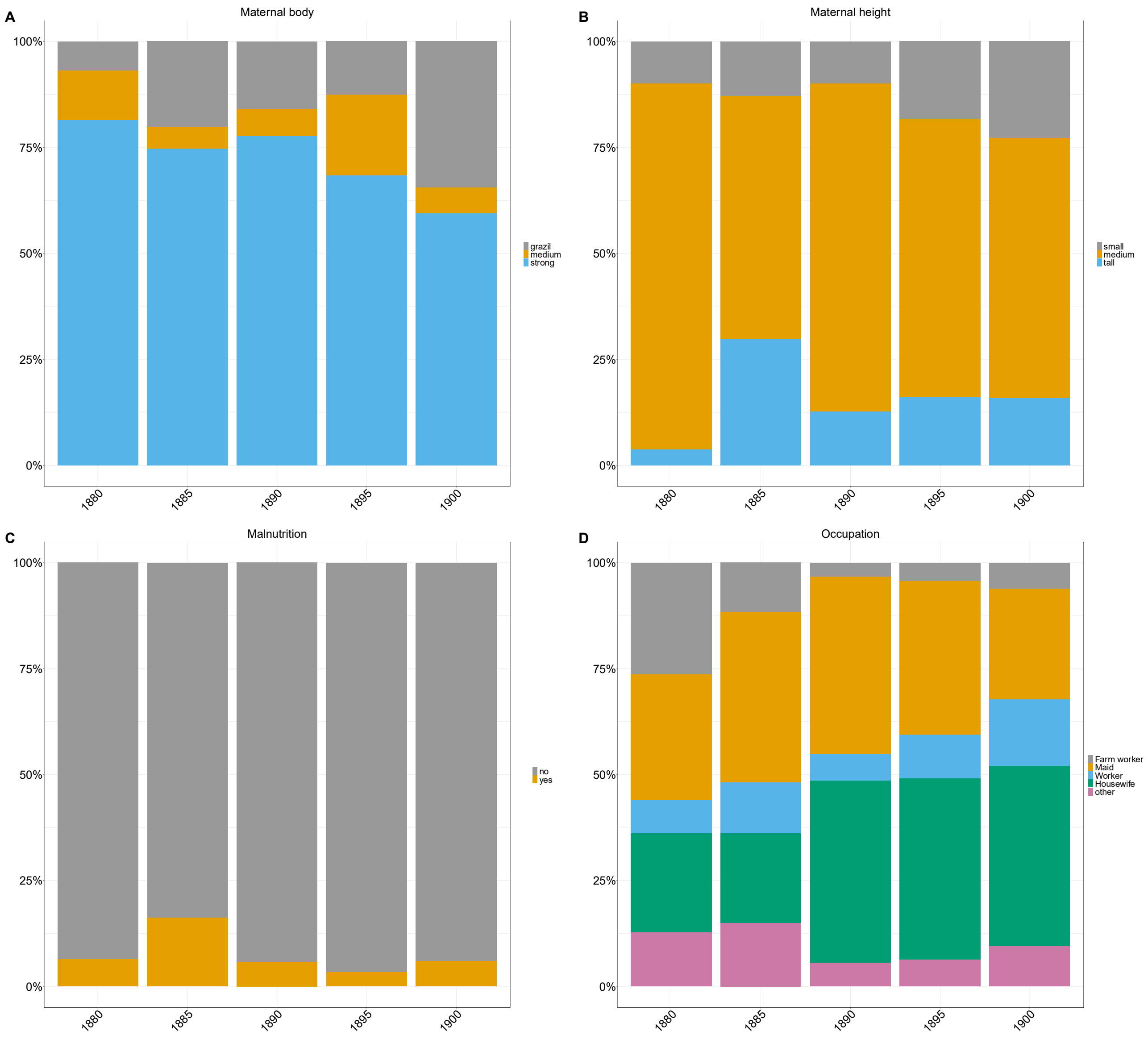

S7 Fig: Time series analysis of birth weight for birth years 1914 – 1922.

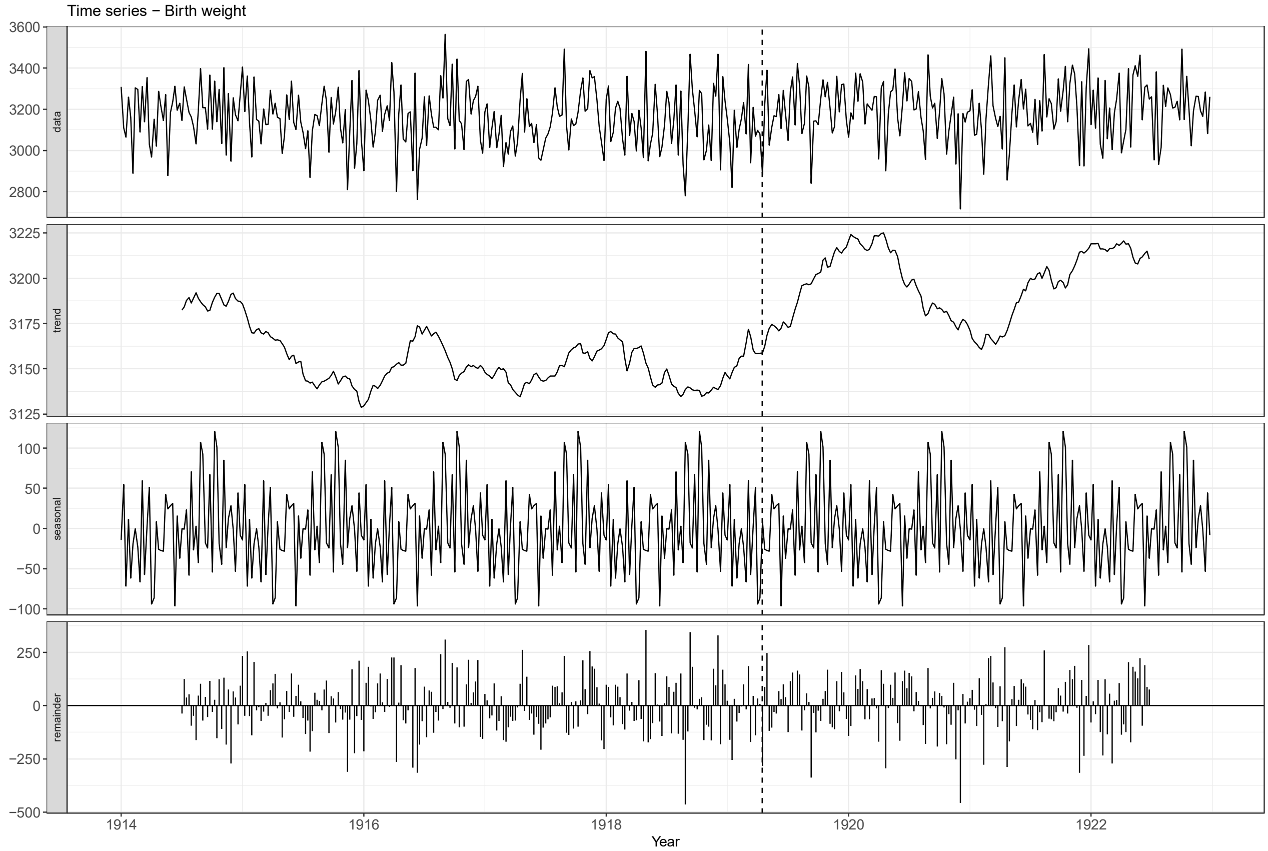

S8 Fig: Time series analysis of stillborn proportion for birth years 1914 – 1922.

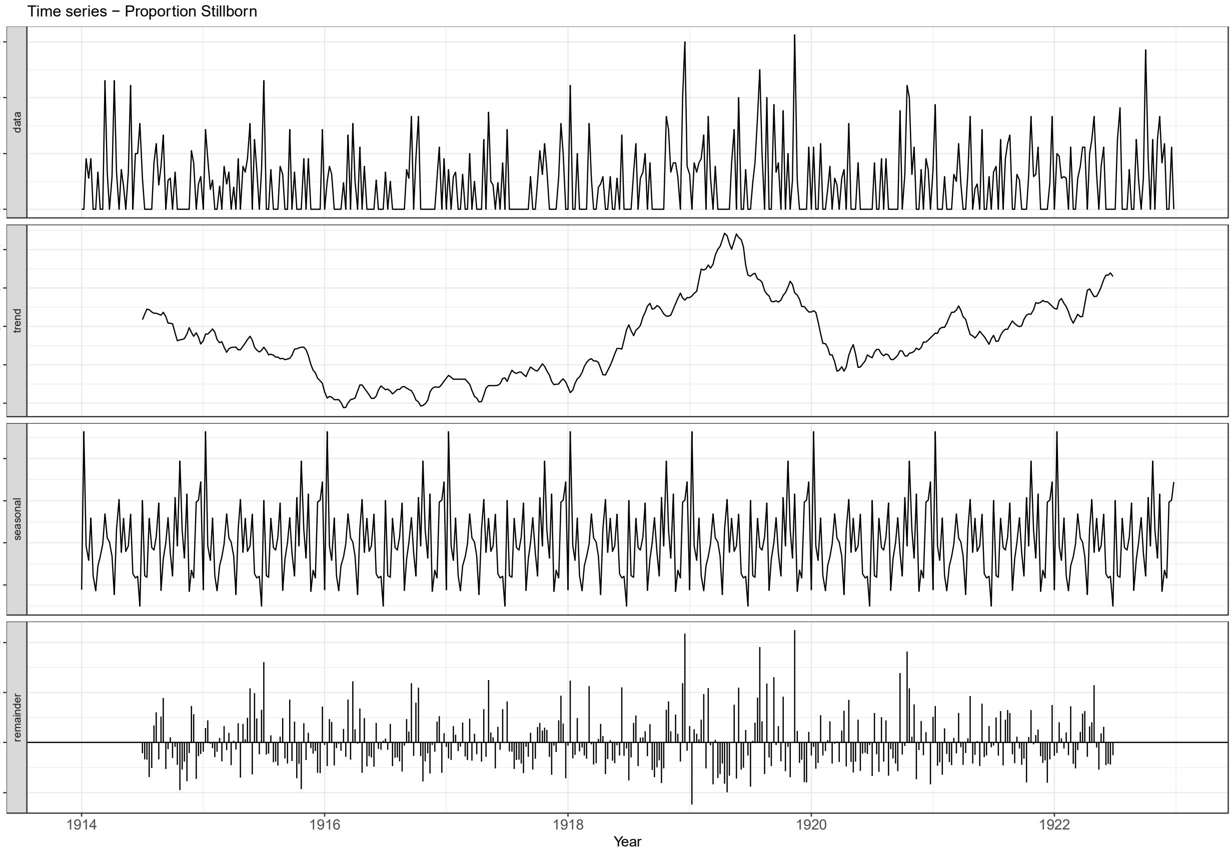

S9 Fig: Time series analysis of gestational weeks for birth years 1914 – 1922.

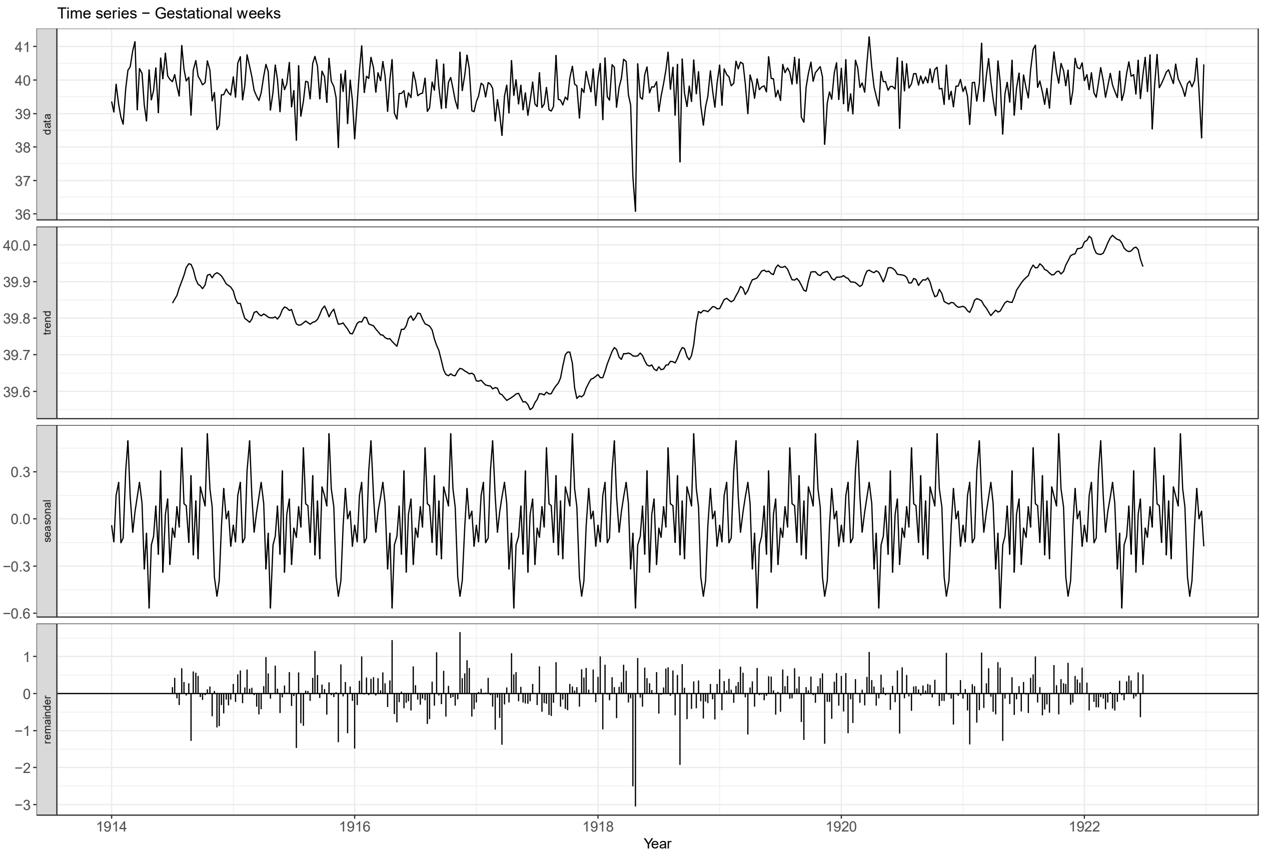

S3 Table: Unadjusted results of linear regression of birth weight for 1880. β describes the regression coefficient and 95%CI the 95% confidence interval.

unadjusted	β (95%CI)
**Year of birth**	
1880*	-
1885	21.74 (-59.93 - 103.41)
1890	-9.07 (-92.77 - 74.62)
1895	-55.18 (-136.79 - 26.42)
1900	26.51 (-53.91 - 106.93)
**Sex**	
Male *	-
Female	-96.44 (-146.8 - -46.09)
**Parity**	
1*	-
2	122.48 (58.2 - 186.75)
3	210.61 (128.93 - 292.29)
>=4	302.91 (240.89 - 364.93)
**Gestational age**	
normal*	-
preterm	-292.78 (-358.36 - -227.2)
**Birthmonth**	
1*	-
2	-69.34 (-191.77 - 53.08)
3	-17.57 (-140.57 - 105.43)
4	23.63 (-100.26 - 147.52)
5	-36.38 (-153.52 - 80.75)
6	-49.77 (-170.33 - 70.79)
7	-66.66 (-192.13 - 58.82)
8	52.77 (-69.1 - 174.65)
9	22.07 (-100.08 - 144.22)
10	85.07 (-43.62 - 213.76)
11	-6.37 (-131.84 - 119.11)
12	115.13 (0.11 - 230.16)
**Urbanity**	
urban*	-
rural	64.41 (9.12 - 119.7)
**Maternal height**	
middle*	-
small	-95.81 (-169.58 - -22.03)
tall	58.08 (-12.23 - 128.39)
**Malnutrition**	
no*	-
yes	-37.3 (-136.04 - 61.45)
**Maternal body**	
normal*	-
grazil	-95.81 (-169.58 - -22.03)
strong	58.08 (-12.23 - 128.39)
**Occupation**	
housewife*	-
farm worker	76.41 (-17.32 - 170.15)
maid	-10.03 (-72.68 - 52.61)
factory worker	-5.87 (-96.96 - 85.21)
other	-141.04 (-235.63 - -46.44)
**Age**	9.6 (5.68 - 13.52)
**Maternal menarche**	8.58 (-2.67 - 19.83)

* Reference

S4 Table: Adjusted results of linear regression of birth weight for 1880 – 1900 adjusted for occupation. The results were mutually adjusted for all variables. β describes the regression coefficient and 95%CI the 95% confidence interval.

**adjusted occupation**	**β (95%CI)**
**Year of birth**	
1880*	-
1885	61.72 (-29.35 - 152.78)
1890	-3.04 (-96.39 - 90.31)
1895	-18.57 (-111.61 - 74.47)
1900	96.48 (2.57 - 190.39)
**Sex**	
Male *	-
Female	-92.57 (-146.61 - -38.52)
**Parity**	
1*	-
2	-
3	-
>=4	-
**Gestational age**	
normal	-
preterm	-259.51 (-332.37 - -186.65)
**Birthmonth**	
1*	-
2	-18.63 (-148.82 - 111.56)
3	-5.94 (-136.44 - 124.55)
4	60.26 (-69.7 - 190.21)
5	-30.05 (-156.3 - 96.21)
6	-20.97 (-151.43 - 109.5)
7	-66.68 (-204.09 - 70.72)
8	-30.88 (-165.26 - 103.49)
9	26.72 (-104.92 - 158.36)
10	82.16 (-51.55 - 215.87)
11	8.35 (-123.25 - 139.94)
12	131.95 (9.91 - 253.99)
**Urbanity**	
urban*	-
rural	24.86 (-36.24 - 85.96)
**Maternal height**	
middle*	-
small	-96.97 (-180.65 - -13.3)
tall	29.56 (-46.06 - 105.17)
**Malnutrition**	
no*	-
yes	6.03 (-105.36 - 117.41)
**Maternal body**	
normal*	-
grazil	-91.08 (-205.84 - 23.69)
strong	27.04 (-69.74 - 123.81)
**Occupation**	
housewife*	-
farm worker	48.13 (-57.07 - 153.33)
maid	6.73 (-61.3 - 74.76)
factory worker	-9.4 (-105.27 - 86.48)
other	-127.19 (-223.23 - -31.15)
**Age**	5.9 (-5.44 - 17.24)
**Maternal menarche**	17.82 (-39.7 - 75.34)

* Reference

S5 Table: Unadjusted results of linear regression of birth weight for 1880. β describes the regression coefficient and 95%CI the 95% confidence interval.

	β (95%CI)
**Year of birth**	
1914*	-
1915	-27.95 (-78.63 - 22.72)
1916	-9.15 (-58.57 - 40.27)
1917	-44.02 (-93.13 - 5.09)
1918	-41.12 (-91.14 - 8.89)
1919	0.46 (-52 - 52.92)
1920	14.62 (-34.87 - 64.11)
1921	17.95 (-31.56 - 67.46)
1922	12.07 (-39.41 - 63.55)
**Sex**	
Male *	-
Female	-146.59 (-170.15 - -123.02)
**Parity**	
1*	-
2	144.97 (113.97 - 175.96)
3	205.08 (166.85 - 243.31)
>=4	236.56 (206.77 - 266.35)
**Gestational age**	
normal*	-
preterm	-388.46 (-420.45 - -356.48)
**Birthmonth**	
1*	-
2	-28.58 (-87.03 - 29.87)
3	-1.94 (-58.66 - 54.78)
4	-21.69 (-79.82 - 36.43)
5	-7.85 (-65.29 - 49.59)
6	10.05 (-48.73 - 68.84)
7	-10.49 (-68.08 - 47.1)
8	24.74 (-34.27 - 83.76)
9	17.77 (-40.38 - 75.92)
10	36.56 (-21.23 - 94.34)
11	23.84 (-34.97 - 82.66)
12	5.94 (-53.46 - 65.35)
**Urbanity**	
urban*	-
rural	31.2 (7.36 - 55.03)
**Married**	
yes*	-
no	-134.17 (-161.02 - -107.32)
**Insurance**	
no*	-
yes	125.8 (70.34 - 181.26)
**Age**	8.89 (7.01 - 10.78)

* Reference

S6 Table: Results of the logistical regression of birth weight. The unadjusted results are the results of the individual unadjusted regression models. Because year and flu intensity are highly correlated, we calculated 2 adjusted models. The adjusted results were mutually adjusted for all variables.

**unadjusted**	**OR (95%CI)**
**Year of birth**	
1914*	-
1915	1.07 (0.7 - 1.65)
1916	1.18 (0.78 - 1.78)
1917	1.45 (0.98 - 2.16)
1918	1.55 (1.04 - 2.3)
1919	1.54 (1.02 - 2.33)
1920	0.97 (0.63 - 1.5)
1921	1.04 (0.68 - 1.6)
1922	1.49 (0.99 - 2.24)
**Flu intensity**	1.53 (0.96 - 2.44)

* Reference

**adjusted Year**	**OR (95%CI)**
**Year of birth**	
1914*	-
1915	1.01 (0.65 - 1.57)
1916	1.16 (0.77 - 1.76)
1917	1.49 (1.00 - 2.21)
1918	1.49 (1.00 - 2.23)
1919	1.55 (1.02 - 2.36)
1920	0.93 (0.61 - 1.44)
1921	1.02 (0.67 - 1.57)
1922	1.45 (0.96 - 2.2)
**Flu intensity**	-
**Sex**	
Male *	-
Female	1.58 (1.3 - 1.91)
**Parity**	
1*	-
2	0.72 (0.55 - 0.93)
3	0.47 (0.33 - 0.69)
>=4	0.51 (0.37 - 0.69)
**Birthmonth**	
1*	-
2	1.38 (0.9 - 2.11)
3	0.82 (0.52 - 1.3)
4	1.26 (0.82 - 1.94)
5	1 (0.64 - 1.56)
6	0.83 (0.52 - 1.34)
7	1.11 (0.72 - 1.71)
8	0.76 (0.47 - 1.23)
9	0.61 (0.37 - 1.01)
10	0.75 (0.46 - 1.2)
11	1.04 (0.66 - 1.63)
12	0.75 (0.46 - 1.23)
**Urbanity**	
urban*	-
rural	0.96 (0.79 - 1.16)
**Married**	
yes*	-
no	1.11 (0.88 - 1.39)
**Insurance**	
no*	-
yes	0.59 (0.35 - 0.99)
**Age**	1.03 (1.01 - 1.05)

**adjusted Flu intensity**	**OR (95%CI)**
**Year of birth**	
1914*	-
1915	-
1916	-
1917	-
1918	-
1919	-
1920	-
1921	-
1922	-
**Flu intensity**	1.44 (0.9 - 2.3)
**Sex**	
Male *	-
Female	1.58 (1.3 - 1.91)
**Parity**	
1*	-
2	0.71 (0.55 - 0.92)
3	0.48 (0.33 - 0.69)
>=4	0.5 (0.37 - 0.69)
**Birthmonth**	
1*	-
2	1.38 (0.9 - 2.11)
3	0.82 (0.52 - 1.3)
4	1.26 (0.82 - 1.94)
5	1 (0.64 - 1.56)
6	0.83 (0.52 - 1.34)
7	1.11 (0.72 - 1.71)
8	0.76 (0.47 - 1.23)
9	0.61 (0.37 - 1.01)
10	0.75 (0.46 - 1.2)
11	1.04 (0.66 - 1.63)
12	0.75 (0.46 - 1.23)
**Urbanity**	
urban*	-
rural	0.96 (0.79 - 1.16)
**Married**	
yes*	-
no	1.1 (0.87 - 1.38)
**Insurance**	
no*	-
yes	0.59 (0.35 - 0.99)
**Age**	1.03 (1.01 - 1.05)

S7 Table: Results of the logistical regression of stillborn. The unadjusted results are the results of the individual unadjusted regression models. Because year and flu intensity are highly correlated, we calculated 2 adjusted models. The adjusted results were mutually adjusted for all variables.

unadjusted	
**Year of birth**	
1914*	-
1915	0.87 (0.54 - 1.42)
1916	0.69 (0.42 - 1.14)
1917	0.75 (0.46 - 1.21)
1918	0.96 (0.6 - 1.54)
1919	1.35 (0.86 - 2.11)
1920	0.82 (0.51 - 1.33)
1921	0.98 (0.62 - 1.55)
1922	1.23 (0.78 - 1.93)
**Flu intensity**	2.02 (1.2 - 3.4)

* Reference

adjusted Year	
**Year of birth**	
1914*	-
1915	0.8 (0.49 - 1.33)
1916	0.66 (0.39 - 1.12)
1917	0.66 (0.4 - 1.09)
1918	0.9 (0.55 - 1.47)
1919	1.39 (0.87 - 2.21)
1920	0.87 (0.53 - 1.43)
1921	0.95 (0.59 - 1.53)
1922	1.21 (0.75 - 1.94)
**Flu intensity**	-
**Sex**	
Male *	-
Female	1.02 (0.81 - 1.29)
**Parity**	
1*	-
2	0.88 (0.62 - 1.26)
3	0.66 (0.41 - 1.06)
>=4	1.13 (0.8 - 1.61)
**Gestational age**	
normal*	-
preterm	3.55 (2.77 - 4.55)
**Birthmonth**	
1*	-
2	0.67 (0.36 - 1.24)
3	0.98 (0.58 - 1.66)
4	0.82 (0.47 - 1.43)
5	1.08 (0.64 - 1.82)
6	0.59 (0.31 - 1.1)
7	0.85 (0.49 - 1.47)
8	0.82 (0.46 - 1.46)
9	0.91 (0.52 - 1.58)
10	1.26 (0.75 - 2.11)
11	0.88 (0.5 - 1.52)
12	0.98 (0.56 - 1.71)
**Urbanity**	
urban*	-
rural	1.24 (0.98 - 1.57)
**Married**	
yes*	-
no	0.95 (0.69 - 1.3)
**Insurance**	
no*	-
yes	0.66 (0.35 - 1.24)
**Age**	1.07 (1.05 - 1.1)

adjusted Flu intensity	
**Year of birth**	
1914*	-
1915	-
1916	-
1917	-
1918	-
1919	-
1920	-
1921	-
1922	-
**Flu intensity**	2.27 (1.32 - 3.9)
**Sex**	
Male *	-
Female	1.01 (0.8 - 1.28)
**Parity**	
1*	-
2	0.88 (0.62 - 1.25)
3	0.65 (0.4 - 1.04)
>=4	1.12 (0.79 - 1.59)
**Gestational age**	
normal*	-
preterm	3.5 (2.73 - 4.48)
**Birthmonth**	
1*	-
2	0.66 (0.36 - 1.23)
3	0.97 (0.57 - 1.65)
4	0.81 (0.47 - 1.42)
5	1.07 (0.63 - 1.8)
6	0.59 (0.31 - 1.1)
7	0.87 (0.5 - 1.5)
8	0.82 (0.46 - 1.46)
9	0.93 (0.54 - 1.63)
10	1.29 (0.77 - 2.16)
11	0.92 (0.53 - 1.6)
12	0.98 (0.56 - 1.71)
**Urbanity**	
urban*	-
rural	1.25 (0.98 - 1.58)
**Married**	
yes*	-
no	0.95 (0.69 - 1.3)
**Insurance**	
no*	-
yes	0.68 (0.36 - 1.28)
**Age**	1.07 (1.05 - 1.1)

S8 Table: Results of the logistical regression of gestational age. The unadjusted results are the results of the individual unadjusted regression models. Because year and flu intensity are highly correlated, we calculated 2 adjusted models. The adjusted results were mutually adjusted for all varia

**unadjusted**	
**Year of birth**	
1914*	-
1915	1.06 (0.79 - 1.42)
1916	1.08 (0.81 - 1.44)
1917	1.49 (1.14 - 1.96)
1918	1.31 (0.99 - 1.73)
1919	0.92 (0.67 - 1.26)
1920	0.9 (0.67 - 1.2)
1921	0.96 (0.72 - 1.29)
1922	0.98 (0.73 - 1.33)
**Flu intensity**	0.69 (0.46 - 1.03)

* Reference

**adjusted Year**	
**Year of birth**	
1914*	-
1915	1.06 (0.79 - 1.42)
1916	1.09 (0.81 - 1.45)
1917	1.54 (1.18 - 2.03)
1918	1.32 (0.99 - 1.75)
1919	0.92 (0.67 - 1.26)
1920	0.9 (0.67 - 1.22)
1921	0.95 (0.71 - 1.28)
1922	1 (0.74 - 1.36)
**Flu intensity**	-
**Sex**	
Male *	-
Female	0.9 (0.79 - 1.03)
**Parity**	
1*	-
2	0.87 (0.72 - 1.05)
3	0.76 (0.59 - 0.98)
>=4	1.09 (0.87 - 1.37)
**Birthmonth**	
1*	-
2	0.74 (0.52 - 1.04)
3	0.95 (0.69 - 1.3)
4	1.2 (0.88 - 1.64)
5	0.87 (0.63 - 1.2)
6	1.04 (0.75 - 1.43)
7	0.92 (0.66 - 1.27)
8	0.77 (0.55 - 1.09)
9	0.65 (0.46 - 0.92)
10	0.69 (0.49 - 0.97)
11	1.26 (0.92 - 1.72)
12	0.82 (0.58 - 1.15)
**Urbanity**	
urban*	-
rural	0.91 (0.79 - 1.04)
**Married**	
yes*	-
no	1.34 (1.14 - 1.58)
**Insurance**	
no*	-
yes	0.84 (0.59 - 1.21)
**Age**	0.99 (0.98 - 1.01)

**adjusted Flu intensity**	
**Year of birth**	
1914*	-
1915	-
1916	-
1917	-
1918	-
1919	-
1920	-
1921	-
1922	-
**Flu intensity**	0.67 (0.45 - 1.00)
**Sex**	
Male *	-
Female	0.91 (0.79 - 1.04)
**Parity**	
1*	-
2	0.87 (0.72 - 1.05)
3	0.77 (0.6 - 1)
>=4	1.1 (0.88 - 1.38)
**Birthmonth**	
1*	-
2	0.73 (0.52 - 1.03)
3	0.95 (0.69 - 1.3)
4	1.22 (0.89 - 1.66)
5	0.88 (0.63 - 1.21)
6	1.05 (0.76 - 1.45)
7	0.91 (0.66 - 1.25)
8	0.78 (0.55 - 1.09)
9	0.64 (0.45 - 0.91)
10	0.69 (0.49 - 0.97)
11	1.24 (0.91 - 1.69)
12	0.82 (0.59 - 1.16)
**Urbanity**	
urban*	-
rural	0.91 (0.79 - 1.04)
**Married**	
yes*	-
no	1.34 (1.13 - 1.58)
**Insurance**	
no*	-
yes	0.83 (0.58 - 1.19)
**Age**	0.99 (0.98 - 1.01)
